# Access barriers to obstetric care at health facilities in sub-Saharan Africa—a systematic review

**DOI:** 10.1186/s13643-017-0503-x

**Published:** 2017-06-06

**Authors:** Minerva Kyei-Nimakoh, Mary Carolan-Olah, Terence V. McCann

**Affiliations:** Disciplines of Nursing and Midwifery, Centre for Chronic Disease, College of Health and Biomedicine, Victoria University, PO Box 14428, Melbourne, Victoria 8001 Australia

**Keywords:** Obstetric care, Maternity care, Access, Barriers, Facility-based deliveries, Maternal deaths, Institutional maternal mortality, Sub-Saharan Africa, Developing countries, Systematic review

## Abstract

**Background:**

Since 2000, the United Nations’ Millennium Development Goals, which included a goal to improve maternal health by the end of 2015, has facilitated significant reductions in maternal morbidity and mortality worldwide. However, despite more focused efforts made especially by low- and middle-income countries, targets were largely unmet in sub-Saharan Africa, where women are plagued by many challenges in seeking obstetric care. The aim of this review was to synthesise literature on barriers to obstetric care at health institutions in sub-Saharan Africa.

**Methods:**

This review was guided by the Preferred Reporting Items for Systematic Reviews and Meta-Analyses (PRISMA) checklist. PubMed, Cumulative Index to Nursing and Allied Health Literature (CINAHL), and Scopus databases were electronically searched to identify studies on barriers to health facility-based obstetric care in sub-Saharan Africa, in English, and dated between 2000 and 2015. Combinations of search terms ‘obstetric care’, ‘access’, ‘barriers’, ‘developing countries’ and ‘sub-Saharan Africa’ were used to locate articles. Quantitative, qualitative and mixed-methods studies were considered. A narrative synthesis approach was employed to synthesise the evidence and explore relationships between included studies.

**Results:**

One hundred and sixty articles met the inclusion criteria. Currently, obstetric care access is hindered by several demand- and supply-side barriers. The principal demand-side barriers identified were limited household resources/income, non-availability of means of transportation, indirect transport costs, a lack of information on health care services/providers, issues related to stigma and women’s self-esteem/assertiveness, a lack of birth preparation, cultural beliefs/practices and ignorance about required obstetric health services. On the supply-side, the most significant barriers were cost of services, physical distance between health facilities and service users’ residence, long waiting times at health facilities, poor staff knowledge and skills, poor referral practices and poor staff interpersonal relationships.

**Conclusion:**

Despite similarities in obstetric care barriers across sub-Saharan Africa, country-specific strategies are required to tackle the challenges mentioned. Governments need to develop strategies to improve healthcare systems and overall socioeconomic status of women, in order to tackle supply- and demand-side access barriers to obstetric care. It is also important that strategies adopted are supported by research evidence appropriate for local conditions. Finally, more research is needed, particularly, with regard to supply-side interventions that may improve the obstetric care experience of pregnant women.

**Systematic review registration:**

PROSPERO 2014 CRD42014015549

**Electronic supplementary material:**

The online version of this article (doi:10.1186/s13643-017-0503-x) contains supplementary material, which is available to authorized users.

## Background

The primary concern of a healthcare system is to efficiently provide evidence-based services that meet the clinical/medical needs of clients, as well as meeting their expectations for receiving good quality care. Ideally, healthcare services should be accessible, available, affordable and acceptable to clients at all times. This is particularly important for vulnerable populations with specific needs, such as pregnant women and their infants. Pregnancy is a particular case in point as it requires defined healthcare services, and at least 15% of all pregnancies (possibly more in sub-Saharan Africa) are complicated by potentially life-threatening conditions [1]. Maternal deaths remain lamentably high, and worldwide, 289,000 women are estimated to have lost their lives to maternal deaths in 2013 alone, approaching two thirds (62%) of these deaths occurred in sub-Saharan Africa. Although complications related to childbirth and pregnancy are not always predictable, most maternal deaths are from preventable or treatable causes [2]. Major contributors to maternal deaths are haemorrhage, hypertensive disorders, sepsis/infections, abortion-related deaths and obstructed labour [3].

Sub-Saharan Africa is currently the world region with the worst maternal health outcomes. In 2013, the maternal mortality ratio (MMR) for this region was 510 per 100,000 live births. By comparison, Eastern Asia, also a developing region, had an MMR of only 95 per 100,000 live births, making it a relatively rare event [2]. High MMRs are not uniformly distributed across sub-Saharan Africa, and it is also unlikely that barriers are evenly distributed. For instance, for every 100,000 live births in 2013, Sierra Leone had an MMR of 1100, Central African Republic had 880, South Sudan had 730, Nigeria had 560, and Ghana had 380 [2]. There is also evidence of substantial disparities in maternal health indicators, by place of residence (rural/urban), socioeconomic status and others, which are indicative of inequities in access to healthcare [2].

The key to averting pregnancy-related ill-health and associated adverse outcomes is ensuring regular and holistic care for women throughout pregnancy and, particularly, during child birth and 24 h after, the critical period with the highest incidence of adverse maternal health events [4]. In order to make such care possible, it is crucial to first identify and eliminate any factors that hinder healthcare access and utilisation. In the presence of adequate healthcare services, there is an opportunity to improve maternal health outcomes [5]. The concept of ‘access’ is better explained using three interrelated dimensions; availability, affordability and acceptability. Hence, efforts aimed at promoting equity of access need to be considered in the wider context of these dimensions. This is because the degree to which a population can gain access to services is not solely dependent on the availability of such services but also on such factors as poverty, organisational and sociocultural barriers [5, 6]. Given the contextual influences on barriers to access, it is best to examine healthcare access from the perspectives of different groups, which may have varying needs, interests and expectations. This notion is reflected in the fact that equity of access may be measured as the availability, utilisation or outcomes of services [5].

There is substantial research and literature on barriers to obstetric care services, as it is an important international public health issue. Similar reviews [7–9] have not specifically focused on demand- and supply-side factors or have more broadly examined facilitators and barriers in low- and middle-income countries [7, 9]. In a systematic review by Bohren and colleagues [9], a qualitative evidence synthesis of factors influencing the decision-making process for place of delivery in low- and middle-income countries was conducted. CA Moyer and A Mustafa [8], on the other hand, focused on only quantitative studies that assessed factors associated with facility-based delivery in sub-Saharan Africa. Other researchers adopted a much broader methodology by including reviews and primary studies, regardless of whether a qualitative or quantitative approach was used [7]. The scope of included studies was, however, global. Given that sub-Saharan Africa bears a disproportionately high burden of adverse obstetric outcomes, particularly maternal deaths [2], it is important to identify and consolidate information on trends contributing to the poor outcomes. This mixed-methods review adds to existing knowledge by examining barriers from a more comprehensive viewpoint of providers and service users. It also includes studies with varied methodological approaches. The aim of this review was to synthesise current evidence on barriers to obstetric care at health institutions in sub-Saharan Africa.

## Methods

In this study, we systematically reviewed published quantitative research on barriers to obstetric care utilisation in sub-Saharan Africa and employed a narrative synthesis approach to summarising the findings. The study protocol was registered in PROSPERO, CRD42014015549 (http://www.crd.york.ac.uk/PROSPERO/display_record.asp?ID=CRD42014015549) and published in systematic reviews [10]. The analytical framework for barriers to healthcare access developed by Jacobs et al. [11] was employed in the data synthesis. The framework has two overarching categories; demand-side and supply-side barriers. Under each of these categories, there are four principal themes with sub-themes—geographic accessibility, availability, affordability and acceptability—which are based on the dimensions of access. Factors identified in the studies were grouped under the themes, and any factors that did not belong to the pre-defined themes (emergent themes) were listed under ‘other barriers’ and examined further. In order to obtain comprehensive data for the review, we considered studies focusing on the perspectives of health workers (supply-side factors) and health service users (demand-side factors). This approach enabled us to capture the multiple factors that may be at play in impeding efficient maternity care usage. Data from the review revealed similarities and differences across countries in the sub-Saharan African region and provided lessons for future policy planning, practice and research.

### Search strategy

We searched the online databases PubMed, CINAHL, and Scopus. Search terms used to locate relevant articles included ‘obstetric care’ with ‘access’, ‘barriers’, ‘developing countries’, ‘pregnancy’, ‘morbidity’, ‘mortality’, ‘haemorrhage’, ‘eclampsia’, ‘sepsis/infection’, ‘obstructed labour’, ‘abortion-related complications’ and ‘sub-Saharan Africa’ (Additional file 1). Articles that met the inclusion criteria were primary research studies that examined barriers to obstetric care, targeted women/service users accessing such care as well as maternity care workers. Studies were also published in a peer reviewed scientific journal in English between 2000 and 2015 and conducted in sub-Saharan Africa. Studies which employed quantitative, qualitative or a mixed-methods design were included. Articles were excluded if the reports were based on secondary data analyses or if data regarding obstetric care barriers is not extractable from the text.

### Study selection

The study selection stage, which involved screening of titles and abstracts and retrieval of full texts, was carried out by one author (MKN). Full-text articles were extracted and assessed against the inclusion and exclusion criteria. Selected full-text articles were re-evaluated for data extraction and assessed for quality.

### Data extraction and evidence synthesis

The quality of included studies was assessed using the mixed methods appraisal tool (MMAT) by Pluye and colleagues [12]. The tool was suited for this review as it was specifically developed for quality appraisal in systematic reviews involving qualitative, quantitative and mixed-methods designs. MMAT is reported to have an inter-rater reliability score ranging from moderate to perfect [13] and has been applied by other researchers [14–16] in mixed-methods systematic reviews. There are five sections in the criteria which include qualitative, randomised controlled, non-randomised, descriptive and mixed-methods studies. Qualitative and quantitative sections have four criteria each, and studies are scored by dividing the number of criteria met by four to arrive at a value ranging from 25 to 100%. For studies with mixed-methods designs, the overall quality score is the lowest score of the study components [17]. All studies were included regardless of their quality ranking since the focus of the review was to examine the context in which barriers to obstetric care occurs (Additional file 2).

Given the range of the outcomes in the studies, identified barriers to obstetric care has been summarised using narrative synthesis. The Popay et al. guidance on the conduct of narrative synthesis in systematic reviews was employed in the synthesis [18]. The elements of the narrative synthesis process are developing a theory, developing a preliminary synthesis, exploring relationships within and between studies, and assessing the robustness of the synthesis. No new theory was developed as part of this review; instead, a pre-existing analytical framework [11] was adopted to facilitate organisation and interpretation of the data. The subsequent step involved the creation of a large table for the extraction of relevant data such as author, year of publication, country, study design, sample characteristics, study objectives, data analysis, major findings and quality assessment (Additional file 3). This important stage was iterative and involved studying the articles, taking notes and making initial comparisons so as to gain familiarisation with the data. Quality appraisal of studies was concomitantly carried out. From the table, the study findings were further examined and using thematic analysis, major themes were coded under pre-defined categories in the analytical framework by Jacobs et al. [11] (Table 1). Emergent themes were also aggregated under the label ‘other barriers’. The underlying methodology applied within the thematic analysis approach was the essentialist or realist method, which is based on the experiences, meanings and the reality of participants [19]. Subsequently, relevant information was drawn out and relationships between the studies were more comprehensively described under the discussion, to enhance interpretation of the review data. Greater attention was paid to differences and similarities between studies as regards the settings, populations, outcomes of interest, methodological approaches and how these might have been reflected in the results. Finally, a critical reflection on the narrative synthesis process was undertaken as regards the quality of the primary studies reviewed and strengths and limitations of this systematic review. In addition, the Preferred Reporting Items for Systematic Reviews and Meta-Analyses (PRISMA) checklist was followed to enhance the quality of reporting [20] (Additional file 4).

**Table 1:** Analytical framework for demand- and supply-side barriers to obstetric care

Demand-side barriers (service users)	Countries of study
Geographic accessibility -Indirect costs to households (transport)^1^ -Means of transport available^2^	Ethiopia^1, 2^; Zimbabwe; Nigeria^2^; Tanzania^1, 2^; Sierra Leone^2^; Malawi^2^; Zambia^1^; Kenya^1, 2^; South Africa^1, 2^; Uganda^2^; Mali^1^; Ghana^1, 2^; Gambia^2^; Burkina Faso^1, 2^; Senegal^2^
Availability of services -Information on health care services/providers^3^ -Health education^4^	Ethiopia^3^; Nigeria^3^; Zimbabwe^3^; Malawi; Kenya^3, 4^; Ghana^3, 4^; Uganda^3, 4^; South Africa^3^; Gambia^3^
Affordability of services -Household resources and willingness to pay^5^ -Opportunity costs (often expressed as being too busy to attend/access services)^6^ Cash flow within society^7^	Nigeria^5, 6^; Ethiopia^5, 6^; Tanzania^5^; Sierra Leone^5, 6^; Burkina Faso^5^; Kenya^5^; Ghana^5^; South Africa^5^; Uganda^5^; Zambia^5^; Mali^5^, Malawi^5^; Democratic Republic of Congo^5^; Angola^5^; Zimbabwe^5^; Cameroon^5^; Gambia^6^
Acceptability of services -Households’ expectations^8^ -Low self-esteem and assertiveness (women’s low status in society and a lack of decision-making autonomy)^9^ -Community and cultural preferences^10^ -Stigma^11^ -Lack of health awareness^12^	Nigeria^9, 10, 12^; Ethiopia^8, 9, 10, 11, 12^; Zimbabwe^10, 11, 12^; Burkina Faso^9, 10^; Tanzania^9, 10, 11, 12^; Malawi^9, 10, 11 12^; Kenya^8, 9, 10, 11, 12^; Zambia^8, 9, 10, 12^; South Africa^9, 11^; Ghana^9, 10, 12^; Uganda^9, 10, 11, 12^; Gambia^9, 12^; Zanbia^9^; Mozambique^10^; Senegal^10^; Angola^9, 10^; Cameroon^10^; Mali^9^; Liberia^9^
Other barriers -Religious affiliation/beliefs^13^ -Lower maternal age (teenage/adolescence)^14^ -Low level of formal education (woman, couple or household head)^15^ -Higher parity^16^ -Fear of surgery, episiotomy, HIV testing or other procedures^17^ -Higher maternal age^18^ -Marital status (married, divorced, separated, single, widowed, polygamous marriage)^19^ -Unintended pregnancy^20^ -Rural residence^21^ -Non-attendance/low attendance of antenatal clinic (as barrier to institutional delivery or postnatal services)^22^ -Agricultural occupations (of women or their partners)^23^ -Household access to telephones or mobile phones^40^ -Lack of birth preparation^41^ -Delayed decision-making within family^42^ -Low media exposure^44^ -Higher levels of household wealth^45^	Nigeria^13, 14, 15, 16, 17, 21^; Ethiopia^13, 14, 15, 16, 18, 19, 20, 21, 22, 23, 41 42, 44^; Burkina Faso^13, 15, 45^; Sierra Leone^15^; Tanzania^14, 15, 16, 18, 22, 41^; Malawi^15, 16, 19, 21, 41, 42^; Ghana^13, 15,16, 17, 20, 22, 41, 44^; Kenya^15, 16, 17, 19, 22, 23, 41, 42^; South Africa^14, 15, 18, 19, 21^; Uganda^15, 16, 21, 22, 41, 42^; Rwanda^15, 16^; Democratic Republic of Congo ^15, 16, 20^; Gambia^41^; Senegal^16, 19^; Zambia^15, 41, 42^; Liberia^17^
Supply-side barriers (maternity care workers/health system factors)	Country
Geographic accessibility -Service location^24^	Nigeria^24^; Ethiopia^24^; Tanzania^24^; Burkina Faso^24^; Malawi^24^; Kenya^24^; Ghana^24^; South Africa^24^; Zambia^24^; Rwanda^24^; Uganda^24^; Burkina Faso^24^; Senegal^24^
Availability of services -Unqualified health workers, staff absenteeism, inadequate staff, opening hours^25^ -Waiting time^26^ -Motivation of staff^27^ -Equipment, drugs and other consumables^28^ -Non-integration of health services^29^ -Lack of opportunity (exclusion from services)^30^ -Late or no referral (Poor referral practices/systems)^31^	Nigeria^25, 26, 27, 28, 31^; Tanzania^25, 26, 27, 28, 31^; Ethiopia^25, 26, 30^; Sierra Leone^30^; Malawi^25, 26, 28, 30^; Kenya^25, 26, 27, 28^; Uganda^25, 28, 30, 31^, Ghana^25, 26, 28, 31^; Gambia^26^; (Burundi and northern Uganda)^25, 27, 28, 31^; Cameroon^22, 28^; South Africa^25, 26^; Zambia^28^
Affordability of services -Costs of services, including informal payments^32^ -Private–public dual practices^33^	Zambia^32^; Ethiopia ^32^; South Africa^32^; Kenya^32^, Malawi^32^; Nigeria^32^; Ghana^32^; Tanzania^32^; Angola^32^; Burkina Faso^32^
Acceptability of services -Complexity of billing system and inability to know prices beforehand^34^ -Staff interpersonal skills, including trust^35^	Ethiopia^35^; Malawi ^35^; Zambia^35^; Ghana^35^; South Africa^35^; Kenya^34, 35^; Democratic Republic of Congo^35^; Uganda^35^; Benin^35^; Nigeria^35^; Tanzania^34, 35^; Liberia^35^
Other barriers -Poor clinical skills/non-adherence to clinical protocol (perceived or experienced)^36^ -Poor staff knowledge about emergency obstetric care and the contents of antenatal care counselling services^37^ -Poor/inadequate facilities/services^38^ -Inadequate/lack of professional development/support (in-service training and supervision); non-availability of guidelines and clinical protocols^39^ -Unsatisfactory quality of care^50^ -Lack of empowerment of health workers to enforce change/decisions^51^	Ethiopia^36, 37, 38, 39, 50^; Zimbabwe; Tanzania^36, 38, 39, 50^; Nigeria^36, 37, 38, 39, 50^; Uganda^36, 38, 39, 50^; Malawi^37, 38, 50, 51^; Kenya^36, 38,^ ^50^; Ghana^36, 37 38, 50^; Cameroon^36, 37, 39^; Senegal^50^; Benin^50^; Gambia^50^; Zambia^38, 39, 50^; Angola^36, 50^; Burkina Faso^50^; South Africa^50^

Adapted from Jacobs et al. (2012) [11]

The numbered superscripts represent pre-identified barriers in the analytical framework and additional ones derived from the review. In the second column, the numbers have been matched against the countries where such barriers were reported

The current review was conducted as part of a PhD (by publication) study by MKN. While MKN conducted the data screening and extraction, quality assessment and data synthesis, she did so under the supervision of MCO and TVM, two experienced researchers. All authors had primary responsibility for the development of research questions and study design, and for the intellectual content of the paper. MCO and TVM provided direction and supervision for all aspects of the work and ensured that questions relating to the accuracy or integrity of the work were investigated and resolved. They critically reviewed the methods and made revisions, as required. They also reviewed and discussed the findings and reviewed and edited each draft of the final document.

## Results

Overall, 2974 references were initially located through searching the databases, and an additional 63 located through other sources such as checking the reference lists of located papers. After exclusion of duplicates, 2766 remained, of which 385 were retrieved for full-text review. Of these, 225 studies were excluded for reasons such as using secondary data, country of study being outside sub-Saharan Africa and having primary outcomes that fell outside the scope of the present review. The number retained for further analysis was 160 as shown in the PRISMA 2009 flow diagram [20] (Additional file 5: Figure S1)).

Based on the MMAT scoring guide, 160 studies were assessed. Of the total number, 87 studies met all the quality criteria (100%) applicable to the study types, 60 studies fulfilled three criteria (75%), 11 fulfilled two (50%) and two met only one (25%) (Additional file 2).

### Characteristics of included studies

Overall, about 51% (*n* = 82) of included studies employed a quantitative design, 28% (*n* = 45) were qualitative and 21% (*n* = 33) were mixed-methods studies. Most of the studies were population- or facility-based cross-sectional surveys, and a few were a combination of both. There were a minority of case-control and cohort studies (Additional file 3). Nearly two thirds (61%) of included studies were conducted in the Eastern African sub-region, about 32% were in Western Africa, 4% were in Southern Africa and 2% in Middle (central) Africa. The studies explored the use of antenatal care, delivery care, postnatal care or a combination of these categories. More than 70% of studies examined access barriers to obstetric care with outcomes relating to the health service users’ perspectives. Study populations identified as service users include females in their reproductive age, pregnant women, postnatal women and, in a few cases, their partners, household heads, mothers-in-law or community leaders. A minority of articles focused on either maternity care workers only or maternity care workers and service users. These studies with a main outcome measure relating to access barriers from maternal healthcare providers’ perspectives assessed the providers’ knowledge and competencies, quality of care, as well as gaps in training and supervision.

### Demand- and supply-side barriers

Overall, the analytical framework by Jacobs and colleagues [11] was a valuable tool for organising the wide range of barriers often encountered by obstetric healthcare service providers and those they serve. The framework captured themes and sub-themes that are generally applicable to most healthcare systems/settings. With regards to the findings of this review, most challenges (sub-themes) that were not captured were those specific to the context of care (such as religious beliefs, requiring permission from family/spouse to seek care, rural residence) or to obstetric healthcare provision (unintended pregnancies, non-attendance/low attendance of antenatal clinic, parity, maternal age). Generally, the framework was suited to this review as it provided an effective means of summarising complex and varied data without losing vital aspects of the information gathered. The results are broadly organised under geographic accessibility, availability, affordability and acceptability of services. The main results are summarised in Table 1.

### Demand-side barriers

Demand-side barriers include factors that influence health service users as individuals, households or at the community level [21] and include geographic accessibility, availability of health services, affordability and acceptability.

#### Geographic accessibility

Under geographic accessibility, two main sub-themes are identified; indirect transport costs and means of transport available. Challenges with geographic access at individual or household levels were reported by several studies as lack of transport/difficulty organising transportation or lack of money for the costs associated with transportation [22–76]. These difficulties may delay or prevent women from seeking appropriate obstetric care, when required.

#### Availability of services

Ideally, health service users should have the opportunity to utilise health care whenever the need arises; hence, the care available must be suited to the needs of clients, provided by qualified personnel, and equipped with relevant supplies [77]. As a demand-side element, availability encompasses information on health care services/providers and health education. Jacobs et al. [11] explain that services such as counselling and provision of consumer information on health services could help address barriers related to availability. Availability barriers were expressed in various forms, such as perceiving health facility-based care which was not different from other options/alternatives [78]; being unaware of care, type and nature of services [22, 28, 47, 78–83] and having limited media exposure [22, 79, 84].

#### Affordability of services

Affordability includes household resources and willingness to pay, opportunity costs (that is, other benefits lost as a result of seeking care) and cash flow within society. Studies which identified demand-side affordability as a barrier reported primarily on household resources and willingness to pay rather than cash flow within society [22, 23, 25, 26, 31, 32, 34, 36–38, 40, 41, 46–48, 50, 52, 61, 66–71, 73–75, 78, 79, 83, 85–112]. Some studies cited reports by pregnant women of being too busy or having no time as reasons for non-use of maternity care services [22, 26, 63, 86, 100, 113, 114], which represent opportunity costs.

#### Acceptability of services

From the service users’ perspectives, acceptability of health services encompasses a range of variables including the households’ expectations, women’s self-esteem and assertiveness, community and cultural preferences, stigma and a lack of health awareness. Household expectations included service users’ minimal expectations of cleanliness and non-interference during labour and delivery at health facilities, ability to meet cultural expectations [30] and a perception that health workers were too busy [91]. Social problems related to women’s low self-esteem and assertiveness were highly reported and manifested in various forms such as the husband’s denial of permission, the need for the husband’s/relative’s permission, women’s low decision-making power/autonomy [22, 23, 27–29, 33, 39, 46, 49, 51, 60, 62, 64, 66, 68, 69, 71, 73, 78–80, 94, 97, 104, 110, 111, 114–122] and delayed/ineffective decision-making within the family [47, 56, 65, 123, 124]. Shyness, fear or shame were also reported [28, 113], particularly among sub-groups like teenagers who were possibly afraid of being reprimanded. In an isolated instance, a study by Anyait et al. [38] indicates that women’s autonomy in decision-making promoted antenatal attendance but reduced the likelihood of delivery in health facilities. Also, Kabakyenga et al. [98] found that decisions made together with a husband increased the use of skilled birth attendants as opposed to women making the decision alone.

Community and cultural preferences [59, 121] covers a range of issues and include the following: home delivery being usual practice or feeling more comfortable at home [25, 27, 33, 46, 48, 54, 59, 80, 85, 113, 114]; cultural beliefs/customs [23, 36, 49–51, 59, 62, 64, 67–69, 71, 75, 78, 108–111, 125]; having relatives nearby to provide closer attention [22, 27, 80]; ethnicity [75, 90, 92, 126, 127]; unwillingness to see a male doctor [23, 57, 128] and not being afforded the freedom to assume preferred birthing positions [43, 51, 66, 104, 110, 129].

Additionally, a lack of health literacy among health service users was found to significantly impede access to obstetric services [53, 66–68, 70, 106, 111, 123, 129–131]. This barrier is often highlighted as a lack of perceived need [23, 45, 86, 121] and may be described in diverse ways, including women reporting an absence of illness or indicating that they are ‘doing fine’ [22, 27, 80, 86, 117, 132]; unexpected labour or being unprepared for birth [25, 27, 36, 39, 42, 45, 47, 55, 63, 64, 75, 80, 83, 85, 98, 100, 107, 117, 132, 133] and being unaware of the need to use available services [30, 33, 46, 86, 87, 95, 96, 100, 113, 134].

Stigma perception is another important sub-theme related to acceptability of health services to pregnant women and households. Link and Phelan [135] explain stigma as a phenomenon which involves labelling, stereotyping, separation, status loss and discrimination. Stigma is often associated with particular groups of people in society who may feel vulnerable or have negative attitudes/perceptions about specific social, medical or other problems. For instance, among adolescents, stigma may lead to fear of disclosing pregnancy [28] or non-use of services as a result of feeling shame [49]. Others include fear of HIV-related stigma in pregnancy [83, 94, 136, 137]. In Ethiopia, Fikre and Demissie [39] reported that the social stigma of being considered weak by family members was an important obstacle to utilising health care facilities for delivery.

#### Other demand-side barriers

Religious reasons for not using obstetric services were cited in some studies [60, 73, 79, 90, 97, 99, 111, 125, 128]. People belonging to Muslim religions [90, 99, 128] and African Traditional Religions (a general term for traditional African beliefs and practices) [79] tended to use services less often. This may be due to cultural restrictions on women within certain households or communities preventing them from leaving their homes or seeking care. Fear of medical procedures such as surgery, episiotomy and blood transfusion hindered access to maternity services in a number of studies [43, 66, 74, 81, 83, 89, 97, 122].

Generally, a low-level of formal education of a woman, husband, couple or household head was a significant barrier to using antenatal, delivery or postnatal services, whereas secondary school and above level of education was associated with better utilisation of maternity services [28, 34, 39, 58, 67, 70, 73, 75, 78–82, 84, 85, 88, 90, 92, 94, 95, 99–101, 103, 106, 108, 113, 117, 124, 127, 130, 132, 134, 138–144].

Several studies showed that obstetric services use decreased with increasing parity, that is, mothers with higher number of children used maternity services less often than nulliparous women and those with two or less children [22, 31, 38, 45, 57, 79, 81–84, 90, 92, 100, 101, 106, 113, 140, 141, 145–148]. Additionally, Nwameme et al. [89] reported that for referred obstetric clients, women with higher parity tended to be associated with greater delays between the time referred and compliance with referral.

Some studies reported that comparatively, older women tended to favour home births and utilised health facilities for birthing less often than younger women [32, 45, 58, 75, 80, 84, 106, 132, 146]. Only a few studies reported lower maternal age or teenage motherhood as a barrier [46, 82, 143]. A woman’s marital status, particularly, divorced, separated, single and widowed marital statuses were reported to be associated with lower or non-use of maternity services [57, 82, 101, 113, 117, 136, 145, 149], possibly due to stigma. Women with unplanned or unintended pregnancies made less use of obstetric care services [79, 84, 95, 99, 100, 148], as did women living in rural areas [39, 45, 58, 80, 98, 101, 118, 150]. Late initiation, fewer or non-attendance of antenatal clinics prior to birthing [46, 79, 80, 83, 94, 98, 100, 117, 130, 132, 142, 148] or not receiving counselling at antenatal clinic on facility birthing during pregnancy [118, 149] were important barriers. Women or their partners who were unemployed [82, 143] or employed in agricultural occupations or whose husbands/partners worked in agriculture were also reported to make less use of maternity care services [94, 134].

### Supply-side barriers (health system factors)

Supply-side barriers are inhibiting factors that function at the service delivery level and are beyond the control of health service users, for instance, inadequate skilled personnel [21]. Similar to demand-side barriers, they include geographic accessibility, availability, affordability and acceptability of health services.

#### Geographic accessibility

Service location was widely reported to deter health facility utilisation, since women were often unwilling or unable to cover the distances required to access services [22, 23, 25, 27, 28, 31–33, 36, 45, 48, 51–54, 57, 58, 64–66, 68, 69, 71, 72, 76, 78, 82, 83, 85, 88, 89, 91, 92, 95, 96, 98, 104, 106, 107, 113, 114, 121, 123, 124, 130, 137, 139, 140, 142, 146, 151–156]. From the health system perspective, this constraint is related to poorly located obstetric health facilities and insufficient number of required facilities.

#### Availability of services

Women who used or intended to use maternity care services faced challenges such as inadequate facility opening hours [25, 157]; non-availability of services [111, 129, 158], poor (perceptions of) providers’ competence or clinical skills [64, 74, 76, 110, 111, 123, 151, 158–160] and knowledge [76, 158, 160–162]; inadequate staffing levels [48, 59, 67, 76, 82, 104, 106–108, 111, 153, 155, 157, 162–165] as well as previous experiences of unskilled birthing care from maternity care providers [29]. Generally, women expect to receive care promptly on reaching a health facility; therefore, long waiting times present a significant challenge to accessing health facility-based services [22, 29, 31, 53, 82, 87, 97, 107, 115, 123, 154, 155]. A shortage or absence of drugs and other essential supplies in health facilities were reported in other studies [31, 48, 56, 67, 76, 111, 115, 150, 155, 157, 163, 165–168], and poor referral practices/systems [104, 105, 159, 165] such as referred clients being transported unaccompanied by healthcare staff [89], lack of feedback mechanisms on referred patients [152], and late or no referral [29] hinder efficient patient care and may result in adverse outcomes.

#### Affordability of services

Costs of services were a deterrent to obstetric care utilisation for some service users [30, 33, 43, 55, 56, 65, 72, 74, 76, 83, 91, 100, 112, 114, 118, 121, 129, 134, 153], as was informal payments for services [110, 155]. Service costs is a particularly significant barrier for poorer rural populations which tend to be socioeconomically disadvantaged across sub-Saharan Africa.

#### Acceptability of services

Jacobs et al. [11] identify factors such as complexity of billing system, inability of patients to know prices beforehand and staff interpersonal skills as supply-side elements under acceptability. Poor staff interpersonal skills, either perceived or from previous experiences [53, 65, 68, 74, 75, 103, 111, 121, 128, 129, 133, 154], impacted negatively on service use. Significant issues included a lack of respect for service users [66, 108, 131, 158, 167, 169, 170], a lack of trust/confidence in health professionals or more trust in alternative care [50, 60–62, 106, 114, 122, 156, 167] and mistreatment by health workers [22, 27, 28, 30, 33, 41–43, 55, 56, 83, 87, 89, 91, 103, 104, 106, 153, 170, 171]. Other negative attitudes of staff, which discourage service users from using maternity services, include a lack of commitment/motivation to work [31, 119, 165, 167], or for instance, expressing a desire to work abroad [152].

#### Other supply-side barriers

Experiences and perceptions of poor quality of health services also presented challenges for women using obstetric care services [22, 25, 43, 45, 57, 63, 69, 71, 72, 97, 100, 110, 119, 129, 144, 150–152, 154, 156, 171, 172]. A lack of supportive care, neglect and poor assessment of labour was reported by Mselle et al. [29] as was a lack of supervision among healthcare workers [29, 44, 160, 167, 173]. Poor staff knowledge about emergency obstetric care (EmOC) and the contents of antenatal care counselling services [173–176], non-availability of guidelines and clinical protocols [160, 163], and inadequate pre-service and in-service training [160, 168] were additionally reported.

Poor/inadequate facilities and infrastructure [62, 67] such as poor laboratory and ambulance services [115], inadequate health facilities providing essential EmOC [29, 65, 134, 150, 152, 172, 176], lack of cleanliness in facilities [31, 155], overcrowding, [44] and an absence of a reliable power [152, 157, 163] and water supply [157, 163] were other challenges encountered.

## Discussion

This review provides a cross-sectional description of published literature on barriers to obstetric care in sub-Saharan Africa between 2000 and 2015. The discussion follows the major themes in the analytical framework employed, which incorporates different dimensions of access and their determinants; geographic accessibility, availability, affordability and acceptability of services [11]. These themes are considered from service–user and service–provider perspectives. The major findings are discussed below and include financial difficulties, transportation-related barriers and sociocultural issues related to service acceptability and availability. Although these barriers are discussed separately, they are not mutually exclusive; hence, interventions have to be considered holistically.

### Affordability of services

Health financing policies have received significant interest in recent years, especially in low- and middle-income countries, in a bid to promote equitable financial access to healthcare services, particularly, for the poor. For instance, different forms of user fee abolition have been implemented in Ghana, Kenya, South Africa and Uganda [177]. Reduction or elimination of user fees for maternity services has been reported to increase utilisation [79, 126]. Notwithstanding, this review indicates that limited household resources/income present a substantial barrier for service users across several sub-Saharan African countries, as is demonstrated in the broader literature [112, 178, 179]. Even in countries where maternity services are free, indirect costs, such as transport, may remain a significant barrier for the poor.

In South Asia, a number of countries including Nepal, India, Bangladesh and Pakistan have implemented cash transfer and voucher schemes, which are demand-side financial interventions, to improve maternity care access [180]. These interventions take the form of cash incentives, vouchers, reimbursement of transport cost or free delivery of services. Apart from India, the schemes were at least partly donor-funded. Generally, increased utilisation of maternity services was observed, despite the country-specific challenges encountered, such as corruption, unclear guidelines and inadequate plans for sustainability [180]. With available empirical evidence from other low- and middle-income countries, cash transfer and voucher schemes could be a feasible system for increasing maternity care utilisation in sub-Saharan Africa. The challenge remains to develop models/schemes which are founded on equity and transparency, and rely on state funds rather than donor funds to ensure sustainability.

### Geographic accessibility

When obstetric complications are present, a delay in reaching and receiving EmOC can contribute to high MMR and perinatal mortality [181, 182]. Limited geographic access to care were linked to physical distance between health facilities and service users’ residence on the supply side and the availability of means of transportation and indirect costs incurred in order to reach the required facility on the demand side. This is similar to findings in related studies [7, 9]. It has been suggested that the minimum acceptable number of EmOC facilities is at least five facilities per 500,000 population (including at least one comprehensive facility); however, in dispersed settlements/populations, the minimum may need to be exceeded [1]. This review showed that while coverage of EmOC services was inadequate in several sub-Saharan African countries, especially in remote/rural areas, poverty remains a major constraint in accessing obstetric care, due to inability to afford transport costs. Some studies indicated that the poorest women travelled longer distances to reach health facilities. According to D Maine [183], the estimated average time between the onset of the most common major obstetric complication (postpartum haemorrhage) and death is 2 h and 12 h for antepartum haemorrhage, in the absence of medical interventions. It is, therefore, recommended that pregnant women should ideally live within 2 h of a basic EmOC facility and within 12 h of a comprehensive care facility [184].

Healthcare planners must bear in mind the nature of the terrain, means of transportation available to women and levels of health facilities when developing strategies to increase obstetric care coverage. There is a growing body of literature using geographic information system as a means to measure accessibility to health care and geographic coverage in sub-Saharan African countries [185–187]; however, most studies are small scale studies, suggesting the need to develop linked national data to support implementation decisions.

### Availability of services

The principal demand-side concern related to health service availability was a lack of information on health care services/providers [22, 78, 79, 81, 84], which limits women’s choices. Knowledge about health services is important in decision-making regarding its utilisation. Hence, this deficit suggests the need for extra efforts to make services known to users, especially, through mass media like radio and television, which is associated with better use of health services in Ghana and Ethiopia [22, 79, 84]. On the supply-side, the most significant barriers were long waiting times at health facilities [22, 29, 31, 81, 87, 97, 115] and, to a lesser extent, poor referral practices [29, 89, 152]. Delays in seeking and receiving obstetric care have been studied extensively and cited frequently. Thaddeus and Maine’s 3-delay model [188] refer to delay in the decision to seek care, delay in arrival at a health facility and delay in the provision of adequate care. The third delay—delay in receiving care upon reaching a health facility—points to gaps in health service delivery [188], as reported by other authors [159, 182, 189]. A systematic review of the third delay reported problems such as under-resourced facilities, non-availability of essential drugs, equipment and blood, inadequate clinical guidelines, shortage of power and water, and referral-related issues such as inadequate emergency transport [181], contributing to adverse maternal outcomes [181, 182].

### Acceptability of services

Acceptability encompasses the degree to which health service provision is responsive to the social and cultural expectations of service users [190]. Although the focus of the review literature varied with regard to this dimension of access, major concerns were a lack of health awareness, issues related to stigma and women’s self-esteem/assertiveness. These factors may be difficult to measure, or even anticipate, and may vary by context. Lack of health awareness was prominent in the review literature and may be summarised as showing ignorance about required obstetric health services, for instance, among some pregnant women, an absence of any physical illness meant no need to use maternity services [22, 86]. Stigma or fear of being shamed has been reported by Moyer and Mustapha [8] in a related study. Data from this review suggest that vulnerable populations, such as those with HIV/AIDS and teenagers/adolescents, require targeted approaches to eliminate stigma during their care. The Convention on Elimination of All Forms of Discrimination against Women adopted in 1979 by the United Nations General Assembly is an important document in the history of women’s rights and opportunities [191]. The convention upholds women’s reproductive health rights and, among other things, seeks to establish women’s equal access to good quality healthcare regardless of individual circumstances. Given that most United Nations member countries have ratified the convention, it is important that maternal health is given the attention it requires to reduce adverse outcomes.

Another key finding of this review was that women’s ability to make decisions regarding healthcare is generally weak, with a reliance on husbands/partners and other family members to make such decisions. Conversely, a study in Nepal shows that couple communication and shared decision-making strategies contribute to improvements in pregnancy health practices [192]. In sub-Saharan Africa, approaches to making obstetric care services more acceptable should involve partners and other influential family members like mothers-in-law and encourage joint decision-making. In addition, women should be made aware of their own health needs and taught assertive skills.

On the supply-side, poor interpersonal relationships by staff and associated lack of confidence/trust in services provided presented the most barrier. Poor staff attitudes, whether perceived or experienced, is known to hinder use of obstetric services [9, 193], as these directly influence perceptions of quality of care.

### Transferability of findings

Central to the context of sub-Saharan African countries is the fact that healthcare systems mostly function within poor-resource settings, which contribute to several inequities in access to maternal healthcare. These inequities partly set the conditions for differing challenges identified as barriers. Given the similarities found, it appears that the results of this current review are transferable across the region, in a broad sense, since major barriers were common to most of the countries of publication. However, differences in cultural perspectives, values, and organisation of individual healthcare systems mean that detailed attention to the divergent ways and settings in which service provision and utilisation occur is essential to ensuring more meaningful outcomes.

## Strengths and limitations

The aim of this review was to synthesise evidence on access barriers from obstetric care provider and service user perspectives. The methodological approach employed was a major strength of the review. By integrating articles with quantitative, qualitative and mixed-methods study designs, a broader cross section of findings was represented. In addition to other factors, quantitative studies were more likely to report socio-demographic-related barriers such as parity, birth order, educational level, place of residence, maternal age and marital status. Qualitative studies provided information on more contextual or descriptive factors such as cultural expectations and beliefs, perceptions, experiences with the health system and health decision-making processes within societies. Similar to the methodology adopted in this review, articles with a mixed-methods approach were more wide-ranging in the scope of obstetric care barriers captured. Generally, the barriers identified within the sub-themes of the analytical framework were from differing study designs rather than a homogeneous source.

Over 70% of studies identified focused on barriers from the perspectives of health service users only. Although the comparatively fewer number of studies on the providers’ perspective may result in less frequently reported health system-related barriers, some were captured through the reports of the service users.

Another limitation of the review should be noted. Data extraction and evidence synthesis in systematic reviews are ideally conducted by at least two reviewers in order to strengthen the reliability of the study outcomes. This is important in systematic reviews of effectiveness; it is critical in qualitative and mixed-method systematic reviews where data extraction and synthesis are even more prone to subjective interpretation. Single-person data extraction and evidence synthesis, as was the case in this study, therefore has implications for the study’s rigour, and the findings reported here must be considered in this context. A team-based process may have affected the reporting of findings in a number of ways, for example by retrieving additional findings, modifying specific findings or changing the relative emphasis on different findings. Nonetheless, efforts were made to ensure that the review process was systematic, with strict adherence to the inclusion and exclusion criteria, as well as the guidelines for quality appraisal for mixed methods [17]. MCO and TVM critically reviewed and discussed the study findings.

Also, the data sources used for this review were limited to primary research published in English and indexed in PubMed, CINAHL, and Scopus databases. Hence, we cannot exclude the possibility that some potentially relevant studies may have been missed as a consequence of being indexed elsewhere or being published in a language other than English. However, the search was comprehensive enough to provide insight into major barriers to obstetric care utilisation in sub-Saharan Africa.

Finally, it is note-worthy that even though an analytical framework provides an easy-to-use tool for analyses, it also has the potential for oversimplification or overfitting of information/data gathered. In order to maximise the technique’s usefulness, an additional generic label—‘other barriers’—was created under supply- and demand-side barriers, respectively, so as to accommodate variables that were not captured under the pre-existing themes.

## Implications for research and practice

Previous reviews have helped consolidate knowledge on common barriers to obstetric care in African countries. Insights from the current review build on that knowledge by drawing attention to the significant number of studies reporting the barriers (and facilitators) to obstetric care utilisation and the possibility that the scope and focus of current research may be skewed. The felt needs of service users need to be prioritised and catered to by service providers and those of service providers addressed by policymakers and employers. However, given the relative dearth of research focusing on supply-side barriers, future research should focus on this issue. In particular, intervention studies may offer stronger evidence on workable implementation strategies and shed more light on supply-side issues that are often reported by service users (such as undignified or disrespectful care) but not by providers. This is because service provision characteristics that are frequently cited by service users as barriers may not be identified by providers and may remain largely unresolved. Providers need to address these issues in the same way they address more common barriers like a lack of equipment and supply or inadequate training. Additional research may help service providers understand or clarify why change is needed and to identify potential strategies for improving women’s experiences and expectations. Intervention studies may also assist the development of tools for the training and evaluation of service providers in this regard.

In this review, we highlight the need for greater attention to address barriers to obstetric services. It appears that a common thread between the deterrents to all the dimension of access is poverty/low socioeconomic status. Sub-Saharan African countries will have to more actively pursue interventions targeted at poorer populations, if MMR and other associated adverse outcomes are to be reduced. Although reduction/elimination of user fees for maternity care is gaining popularity in sub-Saharan Africa [177], more needs to be done to reduce indirect costs of care due to widespread poverty. Innovative approaches, such as cash incentives and voucher schemes discussed earlier, seem promising but may require research to test their feasibility and appropriateness for specific contexts. Development of country-level geospatial data on obstetric facilities and services is critical to improving geographic access, as is regular follow-up audits to reassess population changes and associated shifts in obstetric needs.

Additionally, greater focus on human resources for health as regards their knowledge, practical skills and interpersonal relationships is important, as these affect availability and acceptability of services. It appears that the unequal power dynamics between health service providers and service users negatively impacts interpersonal relationships with clients, which in turn influences clients’ willingness to utilise services or comply with health information. Disrespectful care (perceived or experienced), which was frequently reported across several countries, is an important deterrent for clients requiring obstetric care services. Hence, it is essential to sensitise service providers through more focused training and supervision. Finding appropriate avenues to reach potential service users with information about the nature and purpose of available services is also important, as is maternity care workers’ capacity to provide audience-appropriate information to pregnant women and their partners, mothers-in law, household heads and others who influence decisions related to obstetric care utilisation. Non-formal education may also provide a strategy to strengthen women’s decision-making abilities regarding their health care choices.

Healthcare systems may be made more responsive to the needs of target groups if more emphasis is placed on sociocultural sensitivity in midwifery education. The literature demonstrates that by overlooking women’s preferences and concerns such as freedom to choose birthing positions, having a birth partner or family present, and fear of various hospital procedures, vulnerable groups of women are excluded from accessing care. A more open and receptive approach by care providers may improve the acceptability of services and increase obstetric care utilisation.

## Conclusion

Barriers to obstetric care access are complex and multi-faceted; hence, they require multi-dimensional approaches that take into consideration the needs of service providers and users. Although the barriers are similar across sub-Saharan African countries, variations exist with regards to the nature and extent of the problem. Country-specific strategies are thus needed to tackle the challenges raised.

Governments are best placed to create favourable conditions to raise the status of women and improve their overall socioeconomic well-being. Improved socio-economic status will have multiple effects and is generally associated with an increased ability to afford health services and associated indirect costs such as means of transport, better access to appropriate health information, higher assertiveness, a reduced likelihood to engage in negative sociocultural practices/beliefs and greater acceptability of maternity care.

Lastly, significant investments in healthcare systems, with a focus on improving healthcare infrastructure (obstetric care facilities, good roads, electricity, water supply, communication) and equipment, human resources for health and community level public health education may lead to improved access to obstetric healthcare services. Identifying and exploiting new opportunities for policies that include key perspectives of accessibility, availability, affordability and acceptability of obstetric care will ensure that important viewpoints or concerns are not overlooked.

## Additional files

Additional file 1: PubMed search strategy. Sample search strategy (for PubMed database). (DOC 51 kb)

Additional file 2: Quality assessment using the mixed methods appraisal tool (MMAT). Quality assessment of included studies. (DOC 160 kb)

Additional file 3: Table S1. Characteristics of included studies. Description of eligible/retained studies. (DOC 378 kb)

Additional file 4: PRISMA 2009 Checklist. PRISMA checklist for reporting of systematic review. (DOC 65 kb)

Additional file 5: PRISMS 2009 Flow Diagram. Flow chart of data extraction process. (DOC 64 kb)

## Abbreviations

CINAHL: Cumulative Index to Nursing and Allied Health Literature
EmOC: Emergency obstetric care
MMAT: Mixed methods appraisal tool
MMR: Maternal mortality ratio
PRISMA: Preferred Reporting Items for Systematic Reviews and Meta-Analyses
