# Supplementary material for: Access barriers to obstetric care at health facilities in sub-Saharan Africa—a systematic review
Source: Syst Rev. 2017 Jun 6;6:110. doi: 10.1186/s13643-017-0503-x (PMC5461715; doi:10.1186/s13643-017-0503-x)
Supplement: Supplementary file 1 — PubMed search strategy. Sample search strategy (for PubMed database). (DOC 51 kb) [file 13643_2017_503_MOESM1_ESM.doc]

Pubmed search strategy

| Obstetric care barriers and sub-saharan africa | (obstetric[All Fields] AND barriers[All Fields] AND ("africa south of the sahara"[MeSH Terms] OR ("africa"[All Fields] AND "south"[All Fields] AND "sahara"[All Fields]) OR "africa south of the sahara"[All Fields] OR ("sub"[All Fields] AND "saharan"[All Fields] AND "africa"[All Fields]) OR "sub saharan africa"[All Fields])) AND ("2000/01/01"[PDAT] : "2015/12/31"[PDAT]) |
| --- | --- |
| maternity care barriers and sub saharan africa | (("obstetrics"[MeSH Terms] OR "obstetrics"[All Fields] OR ("maternity"[All Fields] AND "care"[All Fields]) OR "maternity care"[All Fields]) AND barriers[All Fields] AND ("africa south of the sahara"[MeSH Terms] OR ("africa"[All Fields] AND "south"[All Fields] AND "sahara"[All Fields]) OR "africa south of the sahara"[All Fields] OR ("sub"[All Fields] AND "saharan"[All Fields] AND "africa"[All Fields]) OR "sub saharan africa"[All Fields])) AND ("2000/01/01"[PDAT] : "2015/12/31"[PDAT]) |
| maternity care access and sub saharan africa | obstetric[All Fields] AND care[All Fields] AND access[All Fields] AND ("africa south of the sahara"[MeSH Terms] OR ("africa"[All Fields] AND "south"[All Fields] AND "sahara"[All Fields]) OR "africa south of the sahara"[All Fields] OR ("sub"[All Fields] AND "saharan"[All Fields] AND "africa"[All Fields]) OR "sub saharan africa"[All Fields])) AND ("2000/01/01"[PDAT] : "2015/12/31"[PDAT]) |
| Obstetric care access and sub-saharan africa | (obstetric[All Fields] AND care[All Fields] AND access[All Fields] AND ("africa south of the sahara"[MeSH Terms] OR ("africa"[All Fields] AND "south"[All Fields] AND "sahara"[All Fields]) OR "africa south of the sahara"[All Fields] OR ("sub"[All Fields] AND "saharan"[All Fields] AND "africa"[All Fields]) OR "sub saharan africa"[All Fields])) AND ("2000/01/01"[PDAT] : "2015/12/31"[PDAT]) |
| obstetric care access and developing countries | (obstetric[All Fields] AND care[All Fields] AND access[All Fields] AND ("developing countries"[MeSH Terms] OR ("developing"[All Fields] AND "countries"[All Fields]) OR "developing countries"[All Fields])) AND ("2000/01/01"[PDAT] : "2015/12/31"[PDAT]) |
| maternity care access and developing countries | (("obstetrics"[MeSH Terms] OR "obstetrics"[All Fields] OR ("maternity"[All Fields] AND "care"[All Fields]) OR "maternity care"[All Fields]) AND access[All Fields] AND ("developing countries"[MeSH Terms] OR ("developing"[All Fields] AND "countries"[All Fields]) OR "developing countries"[All Fields])) AND ("2000/01/01"[PDAT] : "2015/12/31"[PDAT]) |
| obstetric care barriers and developing countries | (obstetric[All Fields] AND care[All Fields] AND barriers[All Fields] AND ("developing countries"[MeSH Terms] OR ("developing"[All Fields] AND "countries"[All Fields]) OR "developing countries"[All Fields])) AND ("2000/01/01"[PDAT] : "2015/12/31"[PDAT]) |
| maternity care barriers and developing countries | (("obstetrics"[MeSH Terms] OR "obstetrics"[All Fields] OR ("maternity"[All Fields] AND "care"[All Fields]) OR "maternity care"[All Fields]) AND barriers[All Fields] AND ("developing countries"[MeSH Terms] OR ("developing"[All Fields] AND "countries"[All Fields]) OR "developing countries"[All Fields])) AND ("2000/01/01"[PDAT] : "2015/12/31"[PDAT]) |
| place of delivery and sub-saharan africa | (place[All Fields] AND ("delivery, obstetric"[MeSH Terms] OR ("delivery"[All Fields] AND "obstetric"[All Fields]) OR "obstetric delivery"[All Fields] OR "delivery"[All Fields]) AND ("africa south of the sahara"[MeSH Terms] OR ("africa"[All Fields] AND "south"[All Fields] AND "sahara"[All Fields]) OR "africa south of the sahara"[All Fields] OR ("sub"[All Fields] AND "saharan"[All Fields] AND "africa"[All Fields]) OR "sub saharan africa"[All Fields])) AND ("2000/01/01"[PDAT] : "2015/12/31"[PDAT]) |
| place of delivery and barriers and sub-saharan africa | (place[All Fields] AND ("delivery, obstetric"[MeSH Terms] OR ("delivery"[All Fields] AND "obstetric"[All Fields]) OR "obstetric delivery"[All Fields] OR "delivery"[All Fields]) AND barriers[All Fields] AND ("africa south of the sahara"[MeSH Terms] OR ("africa"[All Fields] AND "south"[All Fields] AND "sahara"[All Fields]) OR "africa south of the sahara"[All Fields] OR ("sub"[All Fields] AND "saharan"[All Fields] AND "africa"[All Fields]) OR "sub saharan africa"[All Fields])) AND ("2000/01/01"[PDAT] : "2015/12/31"[PDAT]) |
| place of delivery and developing countries | (place[All Fields] AND ("delivery, obstetric"[MeSH Terms] OR ("delivery"[All Fields] AND "obstetric"[All Fields]) OR "obstetric delivery"[All Fields] OR "delivery"[All Fields]) AND ("developing countries"[MeSH Terms] OR ("developing"[All Fields] AND "countries"[All Fields]) OR "developing countries"[All Fields])) AND ("2000/01/01"[PDAT] : "2015/12/31"[PDAT]) |
| place of delivery and barriers and developing countries | (place[All Fields] AND ("delivery, obstetric"[MeSH Terms] OR ("delivery"[All Fields] AND "obstetric"[All Fields]) OR "obstetric delivery"[All Fields] OR "delivery"[All Fields]) AND barriers[All Fields] AND ("developing countries"[MeSH Terms] OR ("developing"[All Fields] AND "countries"[All Fields]) OR "developing countries"[All Fields])) AND ("2000/01/01"[PDAT] : "2015/12/31"[PDAT]) |
| skilled birth attendance and sub-saharan africa | (skilled[All Fields] AND ("parturition"[MeSH Terms] OR "parturition"[All Fields] OR "birth"[All Fields]) AND attendance[All Fields] AND ("africa south of the sahara"[MeSH Terms] OR ("africa"[All Fields] AND "south"[All Fields] AND "sahara"[All Fields]) OR "africa south of the sahara"[All Fields] OR ("sub"[All Fields] AND "saharan"[All Fields] AND "africa"[All Fields]) OR "sub saharan africa"[All Fields])) AND ("2000/01/01"[PDAT] : "2015/12/31"[PDAT]) |
| obstetric care access and haemorrhage and sub-saharan africa | (obstetric[All Fields] AND care[All Fields] AND access[All Fields] AND ("haemorrhage"[All Fields] OR "hemorrhage"[MeSH Terms] OR "hemorrhage"[All Fields]) AND ("africa south of the sahara"[MeSH Terms] OR ("africa"[All Fields] AND "south"[All Fields] AND "sahara"[All Fields]) OR "africa south of the sahara"[All Fields] OR ("sub"[All Fields] AND "saharan"[All Fields] AND "africa"[All Fields]) OR "sub saharan africa"[All Fields])) AND ("2000/01/01"[PDAT] : "2015/12/31"[PDAT]) |
| access and eclampsia and sub-saharan africa | (access[All Fields] AND ("eclampsia"[MeSH Terms] OR "eclampsia"[All Fields]) AND ("africa south of the sahara"[MeSH Terms] OR ("africa"[All Fields] AND "south"[All Fields] AND "sahara"[All Fields]) OR "africa south of the sahara"[All Fields] OR ("sub"[All Fields] AND "saharan"[All Fields] AND "africa"[All Fields]) OR "sub saharan africa"[All Fields])) AND ("2000/01/01"[PDAT] : "2015/12/31"[PDAT]) |
| obstetric care access and sepsis and sub-saharan africa | (obstetric[All Fields] AND care[All Fields] AND access[All Fields] AND ("sepsis"[MeSH Terms] OR "sepsis"[All Fields]) AND ("africa south of the sahara"[MeSH Terms] OR ("africa"[All Fields] AND "south"[All Fields] AND "sahara"[All Fields]) OR "africa south of the sahara"[All Fields] OR ("sub"[All Fields] AND "saharan"[All Fields] AND "africa"[All Fields]) OR "sub saharan africa"[All Fields])) AND ("2000/01/01"[PDAT] : "2015/12/31"[PDAT]) |
| obstetric care access and obstructed labour and sub-saharan africa | (obstetric[All Fields] AND care[All Fields] AND access[All Fields] AND obstructed[All Fields] AND ("labour"[All Fields] OR "work"[MeSH Terms] OR "work"[All Fields] OR "labor"[All Fields] OR "labor, obstetric"[MeSH Terms] OR ("labor"[All Fields] AND "obstetric"[All Fields]) OR "obstetric labor"[All Fields]) AND ("africa south of the sahara"[MeSH Terms] OR ("africa"[All Fields] AND "south"[All Fields] AND "sahara"[All Fields]) OR "africa south of the sahara"[All Fields] OR ("sub"[All Fields] AND "saharan"[All Fields] AND "africa"[All Fields]) OR "sub saharan africa"[All Fields])) AND ("2000/01/01"[PDAT] : "2015/12/31"[PDAT]) |
| access to abortion care and sub-saharan africa | (access[All Fields] AND ("abortion, induced"[MeSH Terms] OR ("abortion"[All Fields] AND "induced"[All Fields]) OR "induced abortion"[All Fields] OR "abortion"[All Fields]) AND care[All Fields] AND ("africa south of the sahara"[MeSH Terms] OR ("africa"[All Fields] AND "south"[All Fields] AND "sahara"[All Fields]) OR "africa south of the sahara"[All Fields] OR ("sub"[All Fields] AND "saharan"[All Fields] AND "africa"[All Fields]) OR "sub saharan africa"[All Fields])) AND ("2000/01/01"[PDAT] : "2015/12/31"[PDAT]) |
| barriers to safe abortion care in sub-saharan africa | (barriers[All Fields] AND safe[All Fields] AND ("abortion, induced"[MeSH Terms] OR ("abortion"[All Fields] AND "induced"[All Fields]) OR "induced abortion"[All Fields] OR "abortion"[All Fields]) AND care[All Fields] AND ("africa south of the sahara"[MeSH Terms] OR ("africa"[All Fields] AND "south"[All Fields] AND "sahara"[All Fields]) OR "africa south of the sahara"[All Fields] OR ("sub"[All Fields] AND "saharan"[All Fields] AND "africa"[All Fields]) OR "sub saharan africa"[All Fields])) AND ("2000/01/01"[PDAT] : "2015/12/31"[PDAT]) |
| obstetric care barriers maternal morbidity in sub-saharan africa | (obstetric[All Fields] AND care[All Fields] AND barriers[All Fields] AND ("mothers"[MeSH Terms] OR "mothers"[All Fields] OR "maternal"[All Fields]) AND ("epidemiology"[Subheading] OR "epidemiology"[All Fields] OR "morbidity"[All Fields] OR "morbidity"[MeSH Terms]) AND ("africa south of the sahara"[MeSH Terms] OR ("africa"[All Fields] AND "south"[All Fields] AND "sahara"[All Fields]) OR "africa south of the sahara"[All Fields] OR ("sub"[All Fields] AND "saharan"[All Fields] AND "africa"[All Fields]) OR "sub saharan africa"[All Fields])) AND ("2000/01/01"[PDAT] : "2015/12/31"[PDAT]) |
| obstetric care barriers and pregnancy in sub-saharan africa | (obstetric[All Fields] AND care[All Fields] AND barriers[All Fields] AND ("pregnancy"[MeSH Terms] OR "pregnancy"[All Fields]) AND ("africa south of the sahara"[MeSH Terms] OR ("africa"[All Fields] AND "south"[All Fields] AND "sahara"[All Fields]) OR "africa south of the sahara"[All Fields] OR ("sub"[All Fields] AND "saharan"[All Fields] AND "africa"[All Fields]) OR "sub saharan africa"[All Fields])) AND ("2000/01/01"[PDAT] : "2015/12/31"[PDAT]) |
