# Supplementary material for: Access barriers to obstetric care at health facilities in sub-Saharan Africa—a systematic review
Source: Syst Rev. 2017 Jun 6;6:110. doi: 10.1186/s13643-017-0503-x (PMC5461715; doi:10.1186/s13643-017-0503-x)
Supplement: Supplementary file 2 — Quality assessment using the mixed methods appraisal tool (MMAT). Quality assessment of included studies. (DOC 160 kb) [file 13643_2017_503_MOESM2_ESM.doc]

**Quality assessment using the Mixed Methods Appraisal Tool (MMAT)**

1. Qualitative studies

| Qualitative studies | Afari et al. (2013) | Bazzano et al. (2008) | Bedford et al. (2013) | Chapman (2003) | Somé et al. (2014) | Atuoye et al. (2015) | Atuyambe et al. (2009) | Moyer et al. (2013a) | Moyer et al. (2013b) | Cham et al. (2005) | Crissman et al. (2013) | Chi et al. (2015) | Cofie et al. (2015) | Kaye et al. (2014) | Chapman et al. (2003) | Dahlberg et al. (2015) | De Allegri et al. (2015) | Echoka et al. (2014) | Essendi et al. (2015) | Essendi et al. (2010) | Ganle et al. (2014) | Ganle (2015) | Gebrehiwot et al. (2014) | Gebrehiwot et al. (2012) | Grossmann-Kendall et al. (2001) | Lori and Boyle, (2011) | Kawuwa et al. (2007) |
| --- | --- | --- | --- | --- | --- | --- | --- | --- | --- | --- | --- | --- | --- | --- | --- | --- | --- | --- | --- | --- | --- | --- | --- | --- | --- | --- | --- |
| Source of data | 1 | 1 | 1 | 1 | 1 | 1 | 1 | 1 | 1 | 1 | 1 | 1 | 1 | 1 | 1 | 1 | 1 | 1 | 1 | 1 | 1 | 1 | 1 | 1 | 1 | 1 | 1 |
| Methods of analysis | 1 | 1 | 1 | 1 | 1 | 1 | 1 | 1 | 1 | 1 | 1 | 1 | 1 | 1 | 1 | 1 | 1 | 1 | 1 | 1 | 1 | 1 | 1 | 1 | 1 | 1 | 1 |
| Context | 1 | 1 | 1 | 1 | 1 | 1 | 1 | 1 | 1 | 1 | 1 | 1 | 1 | 1 | 1 | 1 | 1 | 1 | 1 | 1 | 1 | 1 | 1 | 1 | 1 | 1 | 1 |
| Reflexivity | 0 | 0 | 0 | 0 | 0 | 0 | 0 | 1 | 1 | 0 | 1 | 0 | 0 | 0 | 0 | 1 | 1 | 0 | 0 | 0 | 1 | 1 | 1 | 1 | 1 | 1 | 0 |
| Overall | 3 | 3 | 3 | 3 | 3 | 3 | 3 | 4 | 4 | 3 | 4 | 3 | 3 | 3 | 3 | 4 | 4 | 3 | 3 | 3 | 4 | 4 | 4 | 4 | 4 | 4 | 3 |

| Qualitative studies | Kaye et al. (2000) | Keri et al. (2010) | Schack et al. (2014) | Seljeskog et al. (2006)  irmeta et al. (2013) | Sialubanje et al. (2015) | Spangler and Bloom. (2010) | King et al. (2015 | Kumbani et al. (2013) | Kwagala (2013) | Magoma et al. (2010) | Mahiti et al. (2015) | Mills and Bertrand (2005) | Mkoka et al. (2014) | Mselle et al. (2013) | Mwangome et al. (2012) | O’Donnell et al. (2014) | Okafor et al. (2014) | Pettersson et al. (2003) | Ridge et al. (2010) | Roro et al. (2014) | Izugbgara et al. (2009) |
| --- | --- | --- | --- | --- | --- | --- | --- | --- | --- | --- | --- | --- | --- | --- | --- | --- | --- | --- | --- | --- | --- |
| Source of data | 1 | 1 | 1 | 1 | 1 | 1 | 1 | 1 | 1 | 1 | 1 | 1 | 1 | 1 | 1 | 1 | 1 | 1 | 1 | 1 | 1 |
| Methods of analysis | 1 | 1 | 1 | 1 | 1 | 1 | 1 | 1 | 1 | 1 | 1 | 1 | 1 | 1 | 1 | 1 | 1 | 1 | 1 | 1 | 1 |
| Context | 1 | 1 | 1 | 1 | 1 | 1 | 1 | 1 | 1 | 1 | 1 | 1 | 1 | 1 | 1 | 1 | 1 | 1 | 1 | 1 | 1 |
| Reflexivity | 0 | 1 | 1 | 1 | 1 | 1 | 1 | 0 | 0 | 1 | 1 | 1 | 1 | 1 | 0 | 0 | 0 | 1 | 1 | 1 | 1 |
| Overall | 3 | 4 | 4 | 4 | 4 | 4 | 4 | 3 | 3 | 4 | 4 | 4 | 4 | 4 | 3 | 3 | 3 | 4 | 4 | 4 | 4 |

1. **Quantitative descriptive studies**

| Quantitative descriptive | Adamu & Salihu, (2002) | Aarnio et al. (2013) | Adewemimo et al. (2013) | Alemayehu and Mekonnen (2015) | Joharifard et al. (2012) | Bayley et al. (2013) | Bayou and Gacho (2013 | Tann et al. (2007) | Teferra et al. (2012) | Chaibva et al. (2009) | De Allegri et al. (2011) | Faye et al. (2011) | Feinstein et al. (2013) | Sakeah et al. (2014) | Habte and Demissie (2015) | Kawakatsu et al. (2014) | Amano et al. (2012) | Anyait et al. (2012) | Azuogu et al. (2011) | Egbewale,and Bamidele, (2009) | Exavery et al. (2014) | Fekede and Gabremariam, (2007) | Fikre and Demissie, (2012) | Groen et al. (2013) | Hagos et al. (2014) | Rockers et al. (2009) | Ikeako et al. (2006) |
| --- | --- | --- | --- | --- | --- | --- | --- | --- | --- | --- | --- | --- | --- | --- | --- | --- | --- | --- | --- | --- | --- | --- | --- | --- | --- | --- | --- |
| sampling strategy | 0 | 1 | 1 | 1 | 0 | 1 | 1 | 1 | 1 | 0 | 1 | 1 | 0 | 1 | 1 | 1 | 1 | 1 | 1 | 1 | 0 | 1 | 1 | 1 | 1 | 0 | 1 |
| representative sample | 0 | 1 | 1 | 1 | 1 | 0 | 1 | 1 | 1 | 0 | 1 | 1 | 1 | 1 | 1 | 1 | 1 | 1 | 1 | 1 | 1 | 1 | 1 | 1 | 1 | 1 | 1 |
| appropriate measurements | 1 | 1 | 1 | 1 | 1 | ` | 1 | 1 | 1 | 1 | 1 | 0 | 1 | 1 | 1 | 1 | 1 | 1 | 1 | 1 | 1 | 1 | 1 | 1 | 1 | 1 | 1 |
| response rate | 1 | 1 | 1 | 0 | 1 | 0 | 1 | 1 | 1 | 1 | 4 | 1 | 1 | 1 | 1 | 1 | 1 | 1 | 1 | 1 | 1 | 1 | 1 | 1 | 1 | 1 | 1 |
| Overall | 2 | 4 | 4 | 3 | 3 | 3 | 4 | 4 | 4 | 2 | 4 | 3 | 3 | 4 | 4 | 4 | 4 | 4 | 4 | 4 | 3 | 4 | 4 | 4 | 4 | 3 | 4 |

| Quantitative descriptive | Tsegay et al. (2013) | Vallières et al. (2013) | Van den Boogaard et al. (2008)  irmeta et al. (2013) | van den Broek et al. (2003) | Wado et al. (2013) | Wanjira et al. (2011) | White et al. (2013) | Wilunda et al. (2013) | Worku et al. (2013a) | Worku et al. (2013B) | Kinuthia et al. (2015) | Jennings et al (2015) | Harfouche et al. 2015 | Wilunda et al. 2015a | Wilunda et al. 201b) | Lakew et al. (2015) | Mazalale et al. (2015) | Semali et al. (2015) | Telfer et al. (2002) | Nakua et al. (2015) | Ntambue et al. (2012) | Silal et al. (2014) | Ono et al. (2013) | Speizer et al. (2014) | Idris et al. (2006) | Oguntunde et al. (2010) | Elembi et al. (2004) |
| --- | --- | --- | --- | --- | --- | --- | --- | --- | --- | --- | --- | --- | --- | --- | --- | --- | --- | --- | --- | --- | --- | --- | --- | --- | --- | --- | --- |
| Sampling strategy | 1 | 1 | 1 | 1 | 1 | 0 | 1 | 1 | 1 | 1 | 1 | 1 | 1 | 1 | 1 | 1 | 1 | 1 | 1 | 1 | 1 | 1 | 0 | 1 | 1 | 1 | 1 |
| Representative sample | 1 | 1 | 0 | 1 | 1 | 0 | 1 | 0 | 1 | 1 | 1 | 1 | 1 | 1 | 0 | 1 | 1 | 1 | 1 | 1 | 1 | 1 | 0 | 1 | 1 | 1 | 1 |
| Appropriate measurements | 1 | 1 | 1 | 1 | 1 | 1 | 1 | 1 | 1 | 1 | 1 | 1 | 1 | 1 | 1 | 1 | 1 | 1 | 1 | 1 | 1 | 1 | 1 | 1 | 1 | 1 | 1 |
| Response rate | 1 | 1 | 1 | 1 | 1 | 1 | 1 | 1 | 1 | 1 | 1 | 1 | 1 | 1 | 1 | 1 | 1 | 1 | 1 | 1 | 1 | 1 | 1 | 1 | 1 | 1 | 1 |
| Overall | 4 | 4 | 3 | 4 | 4 | 2 | 4 | 3 | 4 | 4 | 4 | 3 | 4 | 4 | 3 | 4 | 4 | 4 | 4 | 4 | 4 | 4 | 2 | 4 | 4 | 4 | 4 |

| Quantitative descriptive | Kabakyenga et al. (2011) | Kruk et al. (2010) | Kujawski et al. (2015) | Lerberg et al. (2014) | Ng’anjo Phiri et al. (2014) | Lule et al. (2000) | Mbiza et al. (2014) | Medema-Wijnveen et al. (2012) | ) Mills et al. (2008) | Mpembeni et al. (2007) | Mugweni et al. (2008) | Olusanya et al. 2010 | Onah et al. (2006) | Peltzer et al. (2006) | Peltzer et al. (2005) | Prudhomme O’Meara et al. (2013) |
| --- | --- | --- | --- | --- | --- | --- | --- | --- | --- | --- | --- | --- | --- | --- | --- | --- |
| sampling strategy | **1** | 1 | 0 | 1 | 0 | 1 | 1 | 1 | 1 | 1 | 0 | 0 | 1 | 1 | 0 | 1 |
| representative sample | **1** | 1 | 0 | 0 | 1 | 1 | 1 | 0 | 1 | 1 | 0 | 1 | 1 | 1 | 0 | 1 |
| appropriate measurements | **1** | 1 | 1 | 1 | 1 | 1 | 1 | 1 | 1 | 1 | 1 | 1 | 1 | 1 | 1 | 1 |
| response rate | **1** | 1 | 1 | 1 | 1 | 1 | 1 | 1 | 1 | 1 | 1 | 1 | 1 | 1 | 1 | 1 |
| Overall | **4** | 4 | 2 | 3 | 3 | 4 | 4 | 3 | 4 | 4 | 2 | 3 | 4 | 4 | 2 | 4 |

1. **Quantitative non-randomized studies**

| **Quantitative non-randomized** | Bayu et al. (2015a) | Feyissa and Genemo (2013) | Egbewale,and Bamidele, (2009) | Hounton et al. (2008) | Mengesha et al. (2013) | Spangler et al. (2014) | Trani et al. (2011) | Turan et al. (2012) | Ravit et al. (2015) | Bayu et al. (2015b) | Liambila and Kuria, (2014) | Mirkuzie et al. (2014) | Roka et al. (2013) |  |  |  |  |  |  |  |  |  |  |  |  |  | Storeng et al. (2007) |
| --- | --- | --- | --- | --- | --- | --- | --- | --- | --- | --- | --- | --- | --- | --- | --- | --- | --- | --- | --- | --- | --- | --- | --- | --- | --- | --- | --- |
| selection bias | 1 | 1 | 1 | 1 | 1 | 1 | 1 | 1 | O | 1 | 1 | 1 | 1 |  |  |  |  |  |  |  |  |  |  |  |  |  | 1 |
| appropriate measurements | 1 | 1 | 1 | 1 | 1 | 1 | 1 | 1 | 1 | 1 | 1 | 1 | 1 |  |  |  |  |  |  |  |  |  |  |  |  |  | 1 |
| Comparability of groups | 1 | 1 | 0 | 1 | 1 | 1 | 1 | 1 | 1 | 1 | 1 | 1 | 1 |  |  |  |  |  |  |  |  |  |  |  |  |  | 1 |
| Outcome data | 1 | 1 | 1 | 1 | 1 | 1 | 1 | 1 | 1 | 1 | 1 | 1 | 1 |  |  |  |  |  |  |  |  |  |  |  |  |  | 1 |
| Overall | 4 | 4 | 3 | 4 | 4 | 4 | 4 | 4 | 3 | 4 | 4 | 4 | 4 |  |  |  |  |  |  |  |  |  |  |  |  |  | 4 |

1. **Mixed Methods studies**

| Qualitative component | Anastasi et al (2015) | Austin et al. (2015) | Braddick et al. (2015) | Conrad et al. (2012) | Ijadunola et al. (2010) | Birmeta et al. (2013) | Doctor et al. (2012) | Hailu and Berhe, (2014) | Kyomuhendo (2003) | Tlebere et al. (2007) | Tita et al. (2005) | Silal et al. (2012) | Shiferaw et al. (2013) | Nyango et al. (2010) | Nwameme et al. (2014) | Mwaniki et al. (2002) | Mselle et al. (2011) | Tsawe and Susuman, (2014) | Oiyemhonlan et al. (2013) | Oguntunde et al. (2015 | Pfeiffer and Mwaipopo (2013) | MacKeith et al. (2003) | Asres and Davey, (2015) | Dutamo et al. (2015) | Stekelenburg et al. (2004) | Mrisho et al. (2007) | Sorensen et al. (2011) | Singh et al. (2015) | Osubor et al. (2005) | Storeng et al. (2007) |
| --- | --- | --- | --- | --- | --- | --- | --- | --- | --- | --- | --- | --- | --- | --- | --- | --- | --- | --- | --- | --- | --- | --- | --- | --- | --- | --- | --- | --- | --- | --- |
| Source of data | 1 | 1 | 1 | 1 | 1 | 1 | 1 | 1 | 1 | 1 | 1 | 1 | 1 | 1 | 1 | 1 | 1 | 1 | 1 | 1 | 1 | 1 | 1 | 1 | 1 | 1 | 1 | 1 | 1 | 1 |
| Methods of analysis | 1 | 1 | 1 | 1 | 1 | 1 | 1 | 1 | 1 | 1 | 1 | 1 | 1 | 0 | 1 | 0 | 1 | 1 | 1 | 1 | 1 | 1 | 1 | 1 | 1 | 1 | 1 | 1 | 1 | 1 |
| Context | 1 | 1 | 1 | 1 | 1 | 1 | 1 | 1 | 1 | 1 | 1 | 1 | 1 | 0 | 1 | 0 | 1 | 1 | 1 | 1 | 1 | 1 | 1 | 1 | 1 | 1 | 1 | 1 | 1 | 1 |
| Reflexivity | 0 | 0 | 0 | 0 | 1 | 0 | 1 | 0 | 1 | 0 | 1 | 1 | 0 | 0 | 0 | 0 | 0 | 0 | 0 | 0 | 0 | 0 | 0 | 0 | 0 | 0 | 1 | 1 | 0 | 0 |
| Overall | 3 | 3 | 3 | 3 | 4 | 3 | 4 | 3 | 4 | 3 | 4 | 4 | 3 | 1 | 3 | 1 | 3 | 3 | 3 | 3 | 3 | 3 | 3 | 3 | 3 | 3 | 4 | 4 | 3 | 3 |

| Quantitative descriptive component | Anastasi et al (2015) | Austin et al. (2015) | Braddick et al. (2015) | Conrad et al. (2012) | Ijadunola et al. (2010) | Birmeta et al. (2013) | Doctor et al. (2012) | Hailu and Berhe, 2014 | Kyomuhendo (2003) | Tlebere et al. (2007) | Tita et al. (2005) | Silal et al. (2012) | Shiferaw et al. (2013) | Nyango et al. (2010) | Nwameme et al. (2014) | Mwaniki et al. (2002) | Mselle et al. (2011) | Tsawe and Susuman, (2014) | Oiyemhonlan et al. (2013) | Oguntunde et al. (2015 | Pfeiffer and Mwaipopo (2013 | MacKeith et al. (2003) | Asres and Davey, (2015) | Dutamo et al. (2015) | Stekelenburg et al. (2004) | Mrisho et al. (2007) | Sorensen et al. (2011) | Singh et al. (2015) | Osubor et al. (2005) |  |
| --- | --- | --- | --- | --- | --- | --- | --- | --- | --- | --- | --- | --- | --- | --- | --- | --- | --- | --- | --- | --- | --- | --- | --- | --- | --- | --- | --- | --- | --- | --- |
| Sampling strategy | 1 | 1 | 1 | 0 | 1 | 1 | 1 | 1 | 1 | 1 | 1 | 1 | 0 | 0 | 1 | 1 | 1 | 1 | 0 | 0 | 0 | 0 | 1 | 1 | 0 | 1 | 0 | 1 | 0 |  |
| Representa-tive sample | 1 | 1 | 0 | 1 | 1 | 1 | 1 | 1 | 1 | 0 | 1 | 0 | 1 | 1 | 0 | 0 | 0 | 0 | 0 | 1 | 1 | 1 | 1 | 1 | 1 | 1 | 0 | 1 | 1 |  |
| Appropriate measurement | 1 | 1 | 1 | 1 | 0 | 1 | 1 | 1 | 1 | 1 | 1 | 1 | 1 | 1 | 1 | 1 | 1 | 1 | 1 | 1 | 1 | 1 | 1 | 1 | 1 | 1 | 1 | 1 | 1 |  |
| Response rate | 1 | 1 | 1 | 1 | 1 | 1 | 1 | 1 | 1 | 1 | 1 | 1 | 1 | 1 | 1 | 1 | 1 | 1 | 1 | 1 | 1 | 1 | 1 | 1 | 1 | 1 | 1 | 1 | 1 |  |
| Overall | 4 | 4 | 3 | 3 | 3 | 4 | 4 | 4 | 4 | 3 | 4 | 3 | 3 | 3 | 3 | 3 | 3 | 3 | 2 | 3 | 3 | 3 | 4 | 4 | 3 | 4 | 2 | 4 | 3 |  |

| Mixed methods design | Anastasi et al (2015) | Austin et al. (2015) | Braddick et al. (2015) | Conrad et al. (2012) | Ijadunola et al. (2010) | Birmeta et al. (2013) | Doctor et al. (2012) | Hailu and Berhe, (2014) | Kyomuhendo (2003) | Tlebere et al. (2007) | Tita et al. (2005) | Silal et al. (2012) | Shiferaw et al. (2013) | Nyango et al. (2010) | Nwameme et al. (2014) | Mwaniki et al. (2002) | Mselle et al. (2011) | Tsawe and Susuman, (2014) | Oiyemhonlan et al. (2013) | Oguntunde et al. (2015 | Pfeiffer and Mwaipopo (2013 | MacKeith et al. (2003) | Asres and Davey, (2015) | Dutamo et al. (2015) | Stekelenburg et al. (2004) | Mrisho et al. (2007) | Sorensen et al. (2011) | Singh et al. (2015) | Osubor et al. (2005) | Storeng et al. (2007 |
| --- | --- | --- | --- | --- | --- | --- | --- | --- | --- | --- | --- | --- | --- | --- | --- | --- | --- | --- | --- | --- | --- | --- | --- | --- | --- | --- | --- | --- | --- | --- |
| Relevant design | 1 | 1 | 1 | 1 | 1 | 1 | 1 | 1 | 1 | 1 | 1 | 1 | 1 | 1 | 1 | 1 | 1 | 1 | 1 | 1 | 1 | 1 | 1 | 1 | 1 | 1 | 1 | 1 | 1 | 1 |
| Integration of data | 1 | 1 | 1 | 1 | 1 | 1 | 1 | 1 | 1 | 1 | 1 | 1 | 1 | 0 | 1 | 0 | 1 | 1 | 0 | 1 | 1 | 0 | 1 | 1 | 1 | 1 | 1 | 1 | 1 | 1 |
| Limitations | 0 | 0 | 0 | 1 | 1 | 0 | 0 | 1 | 0 | 0 | 1 | 1 | 0 | 0 | 0 | 0 | 0 | 0 | 0 | 0 | 0 | 0 | 0 | 0 | 0 | 0 | 0 | 1 | 0 | 0 |
| Overall | 2 | 2 | 2 | 3 | 3 | 2 | 2 | 3 | 2 | 2 | 3 | 3 | 2 | 1 | 2 | 1 | 2 | 2 | 1 | 2 | 2 | 1 | 2 | 2 | 2 | 2 | 2 | 3 | 2 | 2 |
