# Supplementary material for: Access barriers to obstetric care at health facilities in sub-Saharan Africa—a systematic review
Source: Syst Rev. 2017 Jun 6;6:110. doi: 10.1186/s13643-017-0503-x (PMC5461715; doi:10.1186/s13643-017-0503-x)
Supplement: Supplementary file 3 — Characteristics of included studies. Description of eligible/retained studies. (DOC 378 kb) [file 13643_2017_503_MOESM3_ESM.doc]

**Additional file 3: Table S1: Characteristics of included studies**

| No | **Author/year** | **Country** | **Study Design and data collection method** | **study population/**  **participants** | **Study objectives** | **Outcomes of interest to the review** | **Quality assessment score (%)** |
| --- | --- | --- | --- | --- | --- | --- | --- |
| 1 | Adamu and Salihu, (2002) | Nigeria | Quantitative study: survey | 107 pregnant women between gestational age range of 8–40 weeks | To identify sociocultural and economic factors that act as barriers to women’s use of antenatal care services and hospital delivery | Economic, cultural and other barriers related to women’s perception of their condition. They include limited financial means, religious reasons, husband’s denial, low health literacy, negligence, ignorance, distance | 50 |
| 2 | Amano et al. (2012) | Ethiopia | Quantitative study: survey | stratified cluster sampling used to select 855 mothers who gave birth 12 months before the study | To determine the level of institutional delivery service utilization and associated factors. | Low educational level, viewing home delivery as usual practice and feeling more comfortable with home delivery, long distance to health facility, labour being urgent, lack of money | 100 |
| 3 | Anyait et al. (2012) | Uganda | Quantitative study: survey | 500 women who had a delivery in the two years before survey. Household socio-economic status was assessed. | To identify the independent predictors of health facility delivery | Being from households of low social economic status, having at least 4 births, limited access to transport. Also, women who made the decision to attend antenatal on their own were more likely to deliver in health facilities but women who had autonomy in deciding on place of delivery were less likely to deliver in health facilities. | 100 |
| 4 | Azuogu et al. (2011) | Nigeria | Quantitative study: survey | 430 women (15 to  49 years) who had carried at least one pregnancy to term in the previous 5 years | To identify factors  affecting utilization of antenatal care services | Low annual household income, low level of couple’s education, perception that antenatal care was not different from facility care, being unaware of antenatal care, distance to facility, cultural beliefs, cost of care, and need for husband’s permission | 100 |
| 5 | Birmeta et al. (2013) | Ethiopia | Mixed methods study: survey and focus group  discussions | 419 women who had given birth in the past three years prior to survey and focus groups | To assess the determinants of maternal health care utilization | Reasons for non-attendance of antenatal clinic include absence of illness, no or little knowledge about antenatal care, being too busy, long waiting times, husband’s disapproval, poor quality of service, long distances.  Reasons for home delivery include having relatives nearby, mistreatment by health workers, trust in traditional birth attendants, transport problems, smooth labour, cost of care, high parity, low literacy status of women, lower average monthly family income, low media exposure, distance to health institutions | 75 |
| 6 | Chaibva et al. (2009) | Zimbabwe | quantitative, non-experimental, descriptive research design | Purposive, non-probability sample of 80 adolescent mothers from the postnatal  wards who had delivered their babies without attending antenatal care. | To identify factors influencing adolescents' non-utilisation of antenatal care services | Fear of disclosing the pregnancy, feeling well and the fact that the baby was kicking, no money to register for antenatal care, limited knowledge about antenatal care and its benefits, no required documents to register for antenatal care, attended clinic with traditional birth attendants and religious factors | 50 |
| 7 | De Allegri et al. (2011) | Burkina Faso | Quantitative: household survey | 435 women who reported a pregnancy in the prior 12 months | To identify determinants of utilisation for antenatal care (ANC) and skilled attendance  at birth after a substantial reduction in user fees | Distance from a health facility, traditional African religion, ethnicity (specifically being Samo or Marka), and higher levels of household wealth were all negatively associated with antenatal care utilisation. | 100 |
| 8 | Doctor et al. (2012) | Nigeria | Mixed methods: survey, key informant interviews and focus group discussions | survey of 6,882 married women, 119 interviews and 95 focus group discussions  with community and local government leaders, traditional birth attendants, women who had attended  maternity services and health care providers. | To determine reasons for low utilisation of antenatal and delivery care among women with recent pregnancies, and the socio-cultural beliefs and practices that influenced them. | Most pregnant women had little or no contact with the health care system for reasons of custom, lack of perceived need, distance, lack of transport, lack of spousal permission, cost and/or unwillingness to see a male doctor, staff shortages, preference for privacy of their home | 75 |
| 9 | Egbewale,and Bamidele, (2009) | Nigeria | Quantitative:  survey | 387 adult women who were pregnant or had a child | To examine current level of utilisation of maternal health care in rural and peri-urban communities | Reasons for irregular or non-attendance of antenatal care included not feeling unwell, lack of finance or being busy  Reasons for non-use of maternity services were lack of finance, perception that is was unnecessary, religious beliefs or being unaware of the need to use those services. | 100 |
| 10 | Ekele and Tunau, (2007) | Nigeria | Quantitative: Longitudinal study | 1,080 pregnant women of low risk who initiated antenatal care at the University Teaching Hospital | To determine the proportion of pregnant women who had antenatal care and delivered in the  hospital and the reasons for delivery elsewhere | Reasons for births outside a health facility were privacy lack of transport during labour, precipitate/‘fast’ labour, husband/in-law’s choice, bad attitude of hospital staff, and cost of hospital delivery. | 75 |
| 11 | Exavery et al. (2014) | Tanzania | Quantitative: survey | 915 women of reproductive age who had given birth in the two years prior to the survey. | To assess facilitators and barriers to institutional delivery in three districts of  Tanzania. | Ethnic background, sudden onset of labour, distance to a health facility, unaffordability of transportation cost, woman’s preference, poor quality of care at the facilities and facility not opened. | 75 |
| 12 | Fekede and Gabremariam, (2007) | Ethiopia | Quantitative: survey | 360 pregnant women in six urban sub-cities | Assess antenatal utilisation and factors associated with non-attendance | Service costs, lack of respect from service providers, long waiting times, lack of knowledge about its importance, and lack of privacy | 100 |
| 13 | Fikre and Demissie, (2012) | Ethiopia | Quantitative: survey | 506 women who gave birth in the last two years | Determine the prevalence of institutional delivery and understand the factors associated with institutional delivery | Rural residence, low educational level, sudden onset of labour, lack of transport facilities, lack of money for transportation and social stigma of being considered as weak by the mother and the mother’s family members for utilising a health facility for delivery. Low decision making power of the women and lack of financial resources or income in the rural mothers | 100 |
| 14 | Groen et al. (2013) | Sierra Leone | Quantitative: household survey | 1,205 females of reproductive age (12–50  years of age) | To describe the current status of access to maternal care, family planning use, and place of delivery. | Financial constraints, having no time, care being unavailable, and difficulty organising transportation | 100 |
| 15 | Hagos et al. (2014) | Ethiopia | Quantitative: survey | 4,949 women who delivered in the two  years preceding the survey in 12 randomly selected villages | To determine the  magnitude and identify factors affecting delivery at health institution in two districts | Lower maternal age, low educational status, lower wealth status, religion, occupation women autonomy, non-attendance of antenatal care services, and number of pregnancies, distance, quality and availability of services and perceived providers’ competence. | 100 |
| 16 | Hailu and Berhe, (2014) | Ethiopia | Mixed methods: survey, focus group discussions and in-depth interviews | 485 mothers selected systematically using multistage sampling technique; men and women (36 discussants in total) and six health extension workers in-depth interviews | To explore the determinants of institutional childbirth service utilization among urban and rural women | Lack of health care information, low decision-making autonomy, low level of education. Reasons for home birth included to get close attention from family, dislike institutional delivery service, feel comfortable when giving birth at home, labour being urgent, husband’s influence, not being sick, other family members influence, bad approach of health workers, health facility being far, previous bad experience, previous home delivery was normal, lack of money for transport, traditional birth attendants being present/available | 75 |
| 17 | Hounton et al. (2008) | Burkina Faso | Quantitative: intervention study | A census of all women aged 12–49 years in two districts and all 43 health facilities in two districts. In one districts, a safe motherhood initiative to improve access to skilled care was implemented. Each health centre in an intervention and comparison district was assessed in terms of staffing and physical functioning. | To evaluate the relationships between accessibility, functioning of health centres and utilisation of delivery care | Distance to health facility, level of education and asset ownership were major determinants of delivery care utilisation, but no association was found between the functioning of health centres and institutional birth rates or births by Caesarean section. | 100 |
| 18 | Ijadunola et al. (2010) | Nigeria | Mixed methods: semi-structured  questionnaire and non-participant observation | Census of all 152 health workers employed in the maternity units of public health facilities offering  maternity care in 5 cities of 2 states and non-participant  observation of maternity staff during antenatal clinic sessions using a structured checklist | To assess knowledge of maternity unit operatives about the concept of emergency obstetric care and investigated the  contents of antenatal care counselling services | Staff had poor knowledge of EmOC and most did not provide specific client-centred messages such as birth preparedness and warning/danger signs of pregnancy and delivery in antenatal care sessions.  Lack of competency-based in-service training programmes | 75 |
| 19 | Ikeako et al. (2006) | Nigeria | Quantitative: survey | 1,095 women who had a delivery in the 3 months preceding the first day of data collection | to determine the current influence of formal maternal education and  other factors on the choice of place of delivery by pregnant women | Low educational status, rural residence, inability to afford cost of care, religious reasons, fear of caesarean delivery, the advice of husband, promptness of care, fear of blood transfusion and privacy. | 100 |
| 20 | Joharifard et al. (2012) | Rwanda | Quantitative: survey | 895 women aged 18–50 who had given birth in the previous three years. | To quantify secular trends in health facility delivery and to identify factors that affect the uptake of intrapartum healthcare services. | Higher parity, low educational status, long distances to facility and a history of an offspring death | 75 |
| 21 | Kabakyenga et al. (2011) | Uganda | Quantitative: survey | 759 women who had delivered within 12 months prior the date of the survey and had complete data on the outcome of interest (assistance by skilled birth  attendant). | To assess the influence of birth preparedness practices and decision-making and assistance by skilled birth attendants | Rural residence, low levels of education, low socio-economic status, long distance to facility, non-attendance of antenatal care and lack of birth preparation. Where the women made the final decision in consultation with their husbands the likelihood of choosing assistance by skilled birth attendants was significantly higher than when women made the decision on location of birth alone. | 100 |
| 22 | Kruk et al. (2010) | Tanzania | Quantitative: survey | 1205 women over the age  of 18 with a delivery within the previous five years | To estimate the contribution of individual and community factors in explaining variation in the use of health facilities for childbirth | Having no health insurance, higher parity, | 100 |
| 23 | Lule et al. (2000) | Malawi | Quantitative: survey | Women aged between 15 and 49 years, who had delivered at least one  child | To determine antenatal attendance and place of delivery of women and how they perceived the quality of health care provided | Family refusal of use of health facility, facilities at the health centre, lack of privacy, lack of drugs, poor ambulance service, poor laboratory services, and long waiting time | 100 |
| 24 | Mbiza et al. (2014) | Malawi (rural) | Quantitative: survey | 240 adolescents pregnant for the first time, between 13 and 19 years and those who delivered their first infant within the previous 6 months | Barriers to health-seeking practices during pregnancy among adolescents | Low decision-making autonomy, low level of education, low health literacy, psychological factors such as shyness, fear, stigma and health service factors such as long distance to the facility, a lack of adolescent friendly services, and inaccessible roads. | 100 |
| 25 | Medema-Wijnveen et al. (2012) | Kenya | Quantitative: survey | 1,777 pregnant women of at least 18 years old in their first 7 months of pregnancy, who were visiting the antenatal care clinic for the first time in their pregnancy and did not know  their current HIV status were recruited at nine governmental health facilities. | To explore relationships between women’s perceptions of HIV-related stigma and their attitudes and intentions regarding facility-based childbirth. | Anticipation of HIV-related stigma, being unmarried, | 75 |
| 26 | Mengesha et al. (2013) | Ethiopia | Quantitative: nested case control study | 1065 mothers with 2^nd^ and 3^rd^ trimester pregnancy (213 cases and 852 controls). | To identify the determinants of skilled attendance for delivery | Low level of education, rural residence, low frequency of antenatal care visits and non-use of family planning services not owning a television. | 100 |
| 27 | Mills et al. (2008) | Ghana | Quantitative: survey | 3,433 women  with pregnancy outcomes in the Kassena-Nankana  district | To assess the factors associated with  the use of health professionals for delivery following implementation of a free obstetric care policy | Higher parity, low educational status, being a practitioner of African traditional religion, low level of autonomy, low socio-economic status, low exposure to mass media (listening to radio, reading newspaper, and watching television), low use of antenatal care, not receiving advise at antenatal clinic to give birth with health professionals, not being that birthing services were free, unplanned pregnancy | 100 |
| 28 | Mpembeni et al. (2007) | Tanzania | Quantitative: survey | A multistage cluster random sampling of 974 women who gave birth within one year prior to survey | Use pattern of maternal health services and determinants of skilled  care during delivery | Long distance to the health facility, low health literacy, low socio-economic status, late initiation of antenatal care and lower than 4 attendances, not receiving advise to give birth in a health facility during antenatal care. | 100 |
| 29 | Mselle et al. (2011) | Tanzania | Mixed methods: in-depth interviews and survey | Sixteen women affected by obstetric fistula who met the  inclusion criteria and 151 women admitted in the fistula wards during the  data collection period | To explore the  birthing experiences of women affected by obstetric fistula  and barriers to accessing adequate quality of care during labour and delivery. | Delays at the health facility, Decisions on where to seek care were most often taken by husbands and mothers-in-law, transportation difficulties, a lack of supportive care, neglect, poor assessment of labour and lack of supervision, unskilled birth care and poor referral routines. | 75 |
| 30 | Mugweni et al. (2008) | Zambia | Quantitative: survey | 80 post-natal women,  who had attended ante-natal clinics | To identify factors contributing to low institutional  deliveries | Women's minimal expectations of cleanliness and non-interference during labour, institutional deliveries' costs, traveling expenses, losing family support and the inability to meet cultural expectations, women's lack of knowledge about danger signs of pregnancy and women's negative perceptions of nurses working at the institutions. | 50 |
| 31 | Mwaniki et al. (2002) | Kenya | Mixed methods study: survey and focus group discussions | 200 mothers with children aged one and below attending child welfare clinic and groups of women from 4 clusters | To determine utilisation of antenatal and maternity services | Higher parity, distance to the facility, lack of transportation, delay in admission at hospital dissatisfaction with services as regards shortage of drugs and essential supplies, lack of commitment by staff, poor quality of food and lack of cleanliness in facilities and lack of money. | 25 |
| 32 | Nwameme et al. (2014) | Ghana | Mixed methods study: survey, in-depth interviews and a facility review checklist | 390 antenatal care  clinic attendees (multiparous women only) and in-depth interviews of principal healthcare personnel | To determine referral options available  to women needing emergency obstetric care, assess constraints faced in accessing obstetric referral system and identify associated drawbacks | Poor referral practices, lack of money, lack of trust in services, poor attitudes of nurses, fear of surgery, distance to referral centres, higher parity, inadequate staff strength, inadequate ambulance services, unavailability  of bed spaces in referral centres, and lack of  feedback from referral centres to care providers at the  periphery. | 75 |
| 33 | Nyango et al. (2010) | Nigeria | Mixed methods study: survey, in-depth interviews | 54 certified Nurse-Midwives working in Primary Health Care clinics | To examine the knowledge and competencies of certified nurse-midwives in the five major areas responsible for maternal mortality | Poor quality care, inadequate knowledge and skills of nurse-midwives in obstetric care, poor referral systems and feedback mechanisms, lack of electricity, job dissatisfaction and inadequate basic EmOC facilities | 25 |
| 34 | Olusanya et al. (2010) | Nigeria | Quantitative: survey | 6,465 mothers attending the Bacille Calmette-Guérin (BCG) immunization clinics in inner-city Lagos | Socio-demographic and obstetric characteristics of  mothers attending the Bacille Calmette-Guérin (BCG) immunization clinics and their association with non-hospital delivery and use of unskilled attendants | Being a teenage mother, Muslim religion, low or middle social class, use of herbal drugs in pregnancy, ethnicity (Yoruba tribe), lack of tertiary education or full-time employment, accommodation with shared sanitation facilities and multiparity. | 75 |
| 35 | Onah et al. (2006) | Nigeria | Quantitative: survey | 1095 women who had delivered within 3 months prior to date of data collection | To identify the factors which influenced choice of place of delivery by pregnant women | Rural residence, Muslim religion, a lack of formal education, low socioeconomic class. | 100 |
| 36 | Peltzer et al. (2006) | South Africa | Quantitative: survey | 870 pregnant women who had delivered before recruited from five PMTCT clinics and surrounding communities. Mothers,  mothers-in-law, husbands or partners of the pregnant women were also recruited | To investigate the utilization of delivery services in the context of PMTCT in a rural community in South Africa | Long distance health facility, transportation cost, having no access to telephone, being an older mother.  Childbirth experiences of the mother or mother-in-law greatly influenced the delivery choices in terms of home delivery | 100 |
| 37 | Peltzer et al. (2005) | South Africa | Quantitative: survey | 186 pregnant women (29.6% HIV positive and  70.4% HIV negative) in four clinics in a rural district | To identify factors influencing the utilisation of Prevention of Mother-to-Child Transmission services in a resource poor setting in South Africa | Transportation difficulties, a lack of means of communication with a health facility, long distance to a health facility, stigma, | 50 |
| 38 | Prudhomme O’Meara et al. (2013) | Kenya | Quantitative: survey | 6,200 pregnant women across six districts | To understand  demand-side factors related to use of antenatal care | Being divorced or separated, working outside the home for wages and having a higher number of children under five years in the household, and limited antenatal care attendance (as a barrier to facility birthing). | 100 |
| 39 | Regassa, (2011) | Ethiopia | Quantitative: survey | 1,094 households from two agro climatic zones | Examine the prevalence and factors associated with antenatal care and postnatal care service  utilisations. | Higher parity, being in polygamous marital relations, unplanned pregnancy, illiteracy, older maternal age women and having limited exposure to media. | 75 |
| 40 | Rockers et al. (2009) | Tanzania | Quantitative: survey  Community-based | 1,204 women who had given birth within five years | To investigate whether the frequency of visits and select characteristics of antenatal care were associated with facility delivery. | Higher parity, higher maternal age, long distance to health facility, low frequency of antenatal care attendance as a barrier to facility birthing). | 75 |
| 41 | Sakeah et al. (2014) | Ghana | Quantitative: survey  Community-based (rural) | 407 households/ women who had ever given birth in the three years prior to the survey | To determine the extent to which community health officer-midwives skilled delivery program  achieved its desired outcomes | Ethnicity (Nankana ethnic group) and women with uneducated husbands were less likely to access skilled attendants at birth. | 100 |
| 42 | Shiferaw et al. (2013) | Ethiopia | Mixed methods: survey, in-depth interviews and focus group discussions. | survey among 15–49 year old women, 3 focus group discussions with women, men and community health workers and in-depth interviews with six  health care providers (physician, nurses, and a health officer) and two traditional birth attendants. | To understand why women might continue to prefer home delivery even  when facility based delivery is available at minimal cost. | Belief that institutional delivery is not necessary and not customary, high cost of birthing services, distance to facility, lack of transportation, requiring permission from husband/family to go for treatment, distrust in service quality. | 75 |
| 43 | Silal et al. (2012) | South Africa | Mixed methods: survey, and in-depth interviews | 1,231 quantitative exit interviews with sixteen qualitative in-depth interviews with women (over 18) in two urban and two rural health sub-districts | explores affordability, availability and  acceptability barriers to obstetric care | Rural women faced the greatest barriers, including longest travel times, highest costs associated with delivery, and lowest levels of service acceptability, relative to urban residents. Negative provider-patient interactions, including staff inattentiveness, turning away women in early labour, shouting at patients, and insensitivity towards those who had experienced stillbirths, also inhibited access and compromised quality of care. | 75 |
| 44 | Spangler and Bloom, (2010) | Tanzania | Mixed methods: survey*, participant observation  and in-depth interviews.  *Survey not included (based on secondary data) | women ages 14 and up with prior childbirth experience as well as adult family members, providers of  childbirth care (facility staff and TBAs), and local health officials. | To examines women’s use of biomedical obstetric care in two rural districts | Distance, lack of decision-making power, affordability/cost of services., abuse, neglect, or humiliation from providers, stigma (relating to ethnicity or family increased susceptibility to substandard treatment), | 100 |
| 45 | Spangler et al. (2014) | Kenya | Quantitative: longitudinal study | 390 pregnant women attending rural antenatal clinics | To compare the use of  PMTCT and maternal health services for all women by HIV status and disclosure category | Women living with HIV who had not disclosed to anyone had the lowest levels of maternity and PMTCT service utilization.  Among HIV positive women, disclosure to a male partner had a particularly strong effect on the use of ARVs for PMTCT. Women’s fears and experiences of HIV-related stigma may be driving decisions to not disclose HIV-positive status and resultant avoidance of health services. | 100 |
| 46 | Tann et al. (2007) | Uganda | Quantitative: community survey | 413 women who reported a pregnancy in the previous five years | To examine the range of antenatal and delivery services received in health care facilities and at home. | Financial and transportation difficulties, low level of education. | 100 |
| 47 | Teferra et al. (2012) | Ethiopia | Quantitative: survey | 371 mothers who gave  birth in the last 12 months | To assess factors affecting institutional delivery service utilization among mothers | Wanting closer attention from family members and relatives during birthing, seeing home birthing as usual practice, unexpected labour, perception of not being sick or having no problem at the time of delivery, family influence, rural residence, older maternal age, low level of education, non-attendance of antenatal care, health illiteracy | 100 |
| 48 | Tita et al. (2005) | Cameroon | Mixed methods: survey and in-depth interviews | 328 health workers providing reproductive health care and a subset for in-depth interviews | To examine the awareness and use of evidence-based reproductive health interventions and associated barriers | Overall, awareness of all four evidence-based interventions was low and for 12 of the 13 interventions, prevalence of awareness was higher than prevalence of use  Barriers to awareness was Deficiencies in education and training, a lack of access to educational resources, a lack of a habit of self-learning.  Barriers to use of interventions were a lack of awareness of interventions, a lack of supplies, negative sociocultural beliefs, financial barriers, shortage of skilled health staff and widespread use of alternative interventions | 100 |
| 49 | Tlebere et al. (2007) | South Africa | Mixed methods: semi-structured  quantitative interviews, qualitative case studies and verbal autopsies | Semi-structured household interviews of 178 women, 57 case studies of women with no antenatal care and/or home birth, and verbal autopsies of maternal and infant deaths, conducted in three diverse sites across the country | To explore factors that  impact utilization of maternal health services | A lack of financial resources for transport, accessibility of health services, in particular, the times services were offered, distance to services, and money needed for travel to  services. Also reported was the negative attitude  of some nurses. | 75 |
| 50 | Trani et al. (2011) | Sierra Leone | Quantitative:  Cross sectional analytic survey | All adults who were identified as being disabled, as well as a control  group of randomly selected non-disabled adults (235 women: 100 women with disabilities, 135 non-disabled women) | To compare health status and access to health care services between disabled and non-disabled people | Inequality in access to care was linked to wealth rather than impairment. | 100 |
| 51 | Tsawe and Susuman, (2014) | South  Africa | Mixed methods: survey and in-depth interviews | 267 female participants and six health workers | To explore the main factors associated with access to and use of maternal health care services | Older maternal age, long distance, financial constraints, being unmarried, teenage (15 – 19 years), low educational level, being unemployed, having no knowledge of the services offered, Staff shortages and longer waiting times | 75 |
| 52 | Tsegay et al. (2013) | Ethiopia | Quantitative: survey | 1113 rural women aged 15–49 years who had given birth at least once in the five years prior to the  survey period. among | To determine the prevalence of maternal  health care utilisation and explore its determinants | Being single or widowed, low level of education, long distance to the health facility cost of services, high parity. Others were “not feeling sick”, “lack of awareness of the benefits”, “feeling shame”, “workload”, “health facility too far away” “easy labour”, “transport problems”. | 100 |
| 53 | Turan et al. (2012) | Kenya | Mixed methods: Prospective cross-sectional survey and in-depth interviews | 1,777 pregnant women with unknown HIV status and a sub-sample of women selected for follow-up (all women who tested HIV positive or were not tested for HIV, and a random sample of HIV-negative women, n = 598); in-depth interviews with community health workers, childbearing women, and family members (n = 48) | To examine the role of women’s perceptions of HIV-related stigma during pregnancy and their subsequent utilization of maternity services. | Negative attitudes about persons living with HIV, low level of education, low household wealth, higher parity. Being in women in agricultural occupations, fewer antenatal clinic attendance. | 100 |
| 54 | Vallières et al. (2013) | Uganda | Quantitative: survey | 392 households with at least one child under the age of  60 months | To examine the association between head of household education level and health seeking behaviours at delivery | Limited or a lack of formal education | 100 |
| 55 | Van den Boogaard et al. (2008) | Zambia | Quantitative: survey | 1413 participants: parous women, their husbands, village headmen and elderly women | To analyse factors that contribute to the choice of traditional birth attendants or skilled birth attendants | Distance to health centre, transportation problems, socio-cultural reasons, when labour progressed fast (birth unpreparedness), and economic reasons. | 75 |
| 56 | van den Broek et al. (2003) | Malawi | Quantitative: survey | women in 20,649 households (total population = 59,248) who delivered in the previous 12 months | To assess pregnancy outcome, maternal mortality and health-seeking behaviour in a rural African  population and to assess the effects on these of women’s education, distance from a health centre and household type. | Low educational level, long distance from the health centre, | 100 |
| 57 | Wado et al. (2013) | Ethiopia | Quantitative: survey | 1370 women of age 15–49 years, with a live birth in the two years before the survey | Unintended pregnancies and the use of maternal  health services | Having an unintended pregnancy, women’s low decision-making autonomy, low level of education, low socioeconomic status, longer travel distance from a health facility, discovery of pregnancy after 12 weeks. | 100 |
| 58 | Wanjira et al. (2011) | Kenya | Quantitative: survey | 409 women who had recently delivered while in the study area | To establish delivery practices and associated factors among mothers seeking child welfare services at selected health facilities | Low level of education, higher parity, low health literacy, perceived delays in being attended to and fear of episiotomy. | 50 |
| 59 | White et al. (2013) | Mali | Quantitative: survey | 317 households: women who had given birth in the previous year, their husbands and their mothers-in-law. | To understand how intra-familial power dynamics and the attitudes of women, their husband and their mother-in-law are associated with maternal health practices. | The preferences and opinions of mothers-in-law in favour of traditional practices | 100 |
| 60 | Wilunda et al. (2013) | Ethiopia | Quantitative: survey | 760 women at discharge from the maternity ward (532 analysed)  Facility-based | Measure equity in utilization of emergency  obstetric care and compare the wealth status of EmOC users with women in the general population. | Poverty, rural residence, transportation costs | 75 |
| 61 | Worku et al. (2013a) | Ethiopia | Quantitative: survey | 1,668 eligible women who gave birth in the last 12 months preceding the study | To assess experiences related to obstetric complication and seeking assistance from a skilled provider among women | Inability to judge the severity of morbidities, distance/transport problems, lack of money/cost considerations and use of traditional options at home. | 100 |
| 62 | Worku et al. (2013b) | Ethiopia | Quantitative: linked facility and population-based survey | 1668 women who had births in the year preceding the survey were selected for analysis | To assess the  effect of individual, communal, and health facility characteristics in the utilisation of antenatal, delivery, and postnatal care by a skilled provider. | Higher birth order, low educational status of women and their husbands, low health literacy, women who belonged to communities with mixed (farming and trading) source of income used skilled attendance and postnatal care more than those who belonged to only farming as the main source of income, non-availability of all the six signal functions in the nearby basic essential obstetric care facility, service costs. | 100 |
| 63 | Kinuthia et al. (2015) | Kenya | Quantitative: survey | Women who had delivered an infant in the previous year were visited  at home in 2011 | To assess correlates of facility delivery among recently pregnant HIV-infected women participating in a community-based survey, and to determine whether these correlates were unique when compared to HIV-uninfected women from the same region. | Cost of services, distance to health facility, fear of harsh treatment by health providers, being HIV-infected with lower socioeconomic status, late initiation of antenatal care (as a barrier to facility delivery), higher parity, fear of HIV testing at health facility, fear of caesarean section, rapid progression of labour, lack of transport, perceptions that facilities were closed at night | 100 |
| 64 | Austin et al. (2015) | Ethiopia | mixed methods: quantitative survey, semi-structured, key informant interviews with providers | Twenty-nine, semi-structured, key informant interviews with providers from an urban referral network and quantitative survey data were collected from 111 providers, | To assess barriers to the provision of emergency obstetric care in Addis Ababa from the perspective of healthcare providers by analysing three factors: implementation of national referral guidelines, staff training, and staff supervision | A lack of transportation and communication infrastructure, overcrowding at the referral hospital, insufficient pre-service and in-service training, and absence of supportive supervision as key barriers to provision of quality emergency obstetric care. | 75 |
| 65 | Ravit et al. (2015) | Mali | Quantitative: case control study | 190 women (95 deceased and 95 near-misses) who underwent caesarean interventions. Data were collected from the health workers and persons who accompanied the woman. | To evaluate the direct and indirect expenses  associated with caesarean interventions performed in  emergency obstetric and neonatal care and the factors associated with these expenses. | Women in the lowest socio-economic group faced barriers related to direct expenses for drugs, treatment and indirect expenses for transport and food. | 75 |
| 66 | Jennings et al. (2015) | Nigeria | Quantitative: survey  Community-based | Head of household and, if applicable, one woman aged 15–49 with a child 23 months or less was randomly selected from household. | To examine if women with limited mobile phone access have differential odds of maternal knowledge and health service utilisation as compared to female mobile phone users | As compared to mobile users, women without mobile phone access had significantly lower odds of antenatal care utilisation, skilled birthing services, and modern contraceptive use after adjusting for demographic characteristics. They also had significantly lower knowledge of maternal danger signs and knowledge of antenatal and skilled delivery care | 75 |
| 67 | Harfouche et al. (2015) | Malawi. | Quantitative: desk review of medical record (program evaluation) | women having caesarean deliveries at Bwaila Hospital | To identify quality indicators of caesarean deliveries and determine their relationship to neonatal and maternal morbidity and mortality | Most delays were attributed to a busy operating theatre and delayed transfer to the operating theatre. Infrastructure and personnel limitations are major barriers to the improvement of quality of caesarean deliveries. | 100 |
| 68 | Wilunda et al. (2015a) | Ethiopia | Quantitative: survey | 500 women aged 15–49 years with a delivery in two years prior to the survey | To determine the coverage of at least four antenatal care visits and delivery by a skilled birth attendant and to identify determinants of utilisation of these services in three districts | Time/distance to the health facility, birth unpreparedness, low socio-economic status, low health literacy, rural residence, higher parity, perception of poor/average quality of care at facilities. | 100 |
| 69 | Wilunda et al. (2015b) | Uganda | Quantitative: review of clinical records, survey and observation | All health facilities in Napak and Moroto districts – review of clinical records and registers, interviewing staff and women attending antenatal and postnatal clinics, and observation. | To establish the availability of maternal and  neonatal healthcare services at different levels of health units; to assess their utilisation; and to determine the quality of services provided. | There were gaps in the availability of essential infrastructure, equipment, supplies, drugs and staff for maternal care, as well as poor quality of care. | 75 |
| 70 | Dutamo et al. (2015) | Ethiopia | Mixed methods study: survey and Focus Group Discussions  Community -based | Survey of 623 women and 4 Focus Group Discussions | To identify the use of maternal health service over the course of pregnancy and child  birth in a comprehensive manner | Perception of being healthy, work overload, feeling shame to attend antenatal care, poor quality of the service, not knowing the importance of antenatal care, cost of services, unintended pregnancy, higher parity, low level of education of woman/partner, low average family monthly income. | 75 |
| 71 | Bayu et al. (2015) | Ethiopia | Quantitative: community-based follow up study (survey) | Face to face interviews of 422 pregnant women in Debre Markos, who were in their second and third trimester of pregnancy at the time of survey | Pregnant women’s preference and factors  associated with institutional delivery service utilisation | Women’s report of labour being accidental, ‘no problem has occurred during labour at home’, higher parity, having no formal education (woman/husband), non-attendance of antenatal care (as barrier to facility birthing). | 100 |
| 72 | Lakew et al. (2015) | Ethiopia | Quantitative: survey | 798 women who gave birth within one year  regardless of their delivery place. | To assess women’s skilled assistance seeking behaviour for pregnancy complications among those who gave birth. | Being unable to understand the seriousness of the complications, viewing facility attendance as unnecessary, family disapproval, low monthly household income (below $US25), limited access to transportation, non-attendance of antenatal care (as barrier to facility birthing), belonging to a a age group below 20 years. | 100 |
| 73 | Mazalale et al. (2015) | Malawi | Quantitative: survey | Women who had completed a pregnancy 12 months prior to the day of the survey and who were within two years postpartum | To identify factors associated with delivery outside a health facility in rural Malawi | Being unmarried, low socioeconomic status, rural residence, having no formal education and being a multigravidae | 100 |
| 74 | Asres and Davey, (2015) | Ethiopia | Mixed methods: survey and key informant interviews | 554 women aged 15-49 years, who had lived for at least 5 years in the study area and had given birth at least once in the 5 years prior to the survey and six key informants who were working in maternal health services in the zone | To assess factors  associated with safe delivery service utilisation among women | Being uneducated, higher parity, previous experience of pregnancy-related complication, poor infrastructure in the health facilities (the rooms are cracked, dirty and  with bad odour, no water supply, a lack of 24-hour electricity supply, no telephone service for emergency calls. Facilities were not adequately equipped with basic obstetric equipment and essential supplies or drugs. | 75 |
| 75 | Semali et al. (2015) | Tanzania | Quantitative: survey | randomly selected 744 households with children aged less than five years | To determine the role of social capital in facilitating health facility delivery. | Belonging to the lowest social capital quintile | 100 |
| 76 | MacKeith et al. (2003) | Zambia | Mixed methods study: survey and focus group discussions | 1210 women who had been  pregnant in the previous two calendar years and four focus groups composed of women with one child, women with more than one child and male partners of women with children | Examined access, coverage and quality of care in these maternity services | Poor quality care (such as reports of having been left alone  for "too long" while in labour), belonging to a poor household, women reporting that they "felt well, non-availability of drugs, unaffordable cost of health services, reports that the labour advanced too quickly, poor equipment supply at facilities, and poor sanitation at facility toilets and bathrooms, poor attitudes of staff members | 50 |
| 77 | Pfeiffer and Mwaipopo (2013) | Tanzania | Mixed methods study: survey, focus group discussions and In-depth interviews | 200 women  (i) who had delivered in a health facility,  and (ii) who delivered with the support of a traditional birth attendant were surveyed, 8 focus-group discussions and In-depth interviews | To describe (1) women’s health-seeking behaviour and experiences regarding their use of antenatal and postnatal care; (2) their rationale behind the choice of place and delivery; and to learn (3) about the use of traditional practices and resources applied by traditional birth attendants and how they can be linked to the biomedical health system. | Having no formal education, being younger (aged 15 to 22)  Birth unpreparedness (‘Unexpected delivery’ ‘realizing it too late’), long waiting time. | 50 |
| 78 | Telfer et al. (2002) | Gambia | Quantitative: survey | 623 women who had recently given birth | Experiences of Mothers with Antenatal, Delivery  and Postpartum Care in Rural Gambia | Reports of being too busy, feeling unwell, being away or travelled, inadequate health information provided at antenatal care | 100 |
| 79 | Liambila and Kuria, (2014) | Kenya. | Quantitative: case–control study | women aged 15–49 years at the households (294 cases and 291 controls) | to assess the nature of childbirth related complications among the skilled and the non-skilled birth attendants in  Western Kenya. | Undignified care, high service and transport costs, fear of hospital procedures such as HIV tests, undergoing unnecessary caesarean-section and mishandling of the placenta, poor quality of care (such as limited flexibility to choose birthing position, being taken care of by trainee nurses or students and lack of follow up by health facility staff). expectation to buy supplies such as cotton wool and gloves, not having the option to choose trusted persons to assist them during childbirth (one is assisted by any staff on duty), lack of services such food, warm beverages, and bath water in health facilities. Cultural perceptions that home birth gives a woman more dignity and is a sign that she is strong. | 100 |
| 80 | Mirkuzie et al. (2014) | Ethiopia | Quantitative: facility based intervention | 10 randomly selected public health centers | To examine progress in the implementation of the basic emergency obstetric care in Addis Ababa and compared with the 2008 survey. | Insufficient knowledge in diagnosing postpartum haemorrhage and birth asphyxia as well as poor skills in neonatal resuscitation | 100 |
| 81 | Nakua et al. (2015) | Ghana | Quantitative: survey | Women attending post-partum care with children under-12 months | To assess the effect of an intervention addressing barriers in access to skilled obstetric care and identified factors associated with the use of unskilled birth attendants during delivery in a rural district of Ghana. | Use of insulting language by health workers, unavailability of transport, and confidence in traditional birth attendants, sudden labour, lack of partner involvement, lack of birth preparedness and lack of knowledge of the benefits of skilled delivery. | 100 |
| 82 | Ntambue et al. (2012) | Democratic  Republic of Congo | Quantitative: survey | 1762 women residing in Lubumbashi who had delivered during the 12 months prior to the survey | To determine the factors that influence the  use of mother and child healthcare services in Lubumbashi, Democratic Republic of the Congo | Being a primiparous or grand multiparous woman, having an unplanned pregnancy | 100 |
| 83 | Oguntunde et al. (2015) | Nigeria | Mixed methods: survey, in-depth interviews and an inventory of equipment and supply in facilities | 80 health facilities (80 service providers were interviewed), in-depth interviews with facility managers and an inventory of equipment and supply  in facilities | To examine facilitators and barriers to the use of magnesium sulphate in the management of pre-eclampsia/eclampsia in health facilities in Bauchi and Sokoto States in Nigeria. | Inadequate numbers of skilled providers, frequent shortages of magnesium sulphate, lack of essential equipment and supplies, irregular supply of electricity and water, and non-availability of guidelines and clinical protocols at the health facilities. Technical support to providers was inadequate. | 75 |
| 84 | Silal et al. (2014) | South Africa | Quantitative: records review and survey | 1,491 households with a woman over 18 years of age who had delivered in the preceding year and patient exit interview surveys | To assess the relative socio-economic inequalities in use  of hospital-based maternal delivery services within two rural sub-districts of South Africa. | Long distance to facilities, poor access to ambulance services, high health expenses (transport, supplies, food, and childcare), poor health worker interpersonal relationships with clients (too busy to listen to their problems, show of disrespect) | 100 |
| 85 | Aarnio et al. (2013) | Malawi | Mixed methods study: cross-sectional survey with qualitative component (type not specified) | 389 ever married men whose wives were of reproductive age (15–49 years) and had been pregnant within the last 5 years | Explore how husbands perceive delivery care in rural Malawi. | Cost of services, long distance/transportation, birth unpreparedness, lack of knowledge about types of services offered, delayed decision-making within the family. | 100 |
| 86 | Adewemimo et al. (2014) | Nigeria | Quantitative: cross-sectional survey | 400 women of reproductive age (15–49 years) who had given birth in the last two years preceding the study. | To determine the level and determinants for utilisation of skilled birth attendance | Lack of healthcare providers in the facility, lack of equipment and supplies, poverty, preference for home births, cost of care, long distances, transportation. | 100 |
| 87 | Afari et al. (2013) | Ghana | Qualitative study: semi structured  interviews | Healthcare workerss (8 midwives, 4 community health officers, 3 medical assistants, 2 emergency room nurses, 1 doctor) at different facility levels within the district | To describe healthcare worker identified system-based bottlenecks and the value of local engagement in designing strategies to improve referral processes related to emergency obstetric care  in rural Ghana. | Poor referral transport system, inadequate communication systems, poor clinical skills, non-adherence to clinical protocols, and poor documentation | 75 |
| 88 | Alemayehu and Mekonnen (2015) | Ethiopia | Quantitative: cross-sectional survey | 373 women who had delivered in 12  months prior to the study | To assess the prevalence of skilled birth attendant utilization and its correlates in North West Ethiopia | Low educational status, non-attendance of antenatal care, long distances | 75 |
| 89 | Anastasi et al (2015) | Uganda | Mixed methods:  Structured and semistructured interviews, focus group discussions | Structured antenatal care client entry and exit interviews [n = 139]; semi-structured interviews with women in their homes  [n = 36], health workers [n = 10], and policymakers [n = 10]; and focus group discussions with women [n = 20], men [n = 20], and traditional birth attendants [n = 20]. | To identify key factors underlying the gap between high rates of antenatal care attendance and much lower rates of health-facility delivery; examine the association between advice during antenatal care to deliver  at a health facility and actual place of delivery; investigate whether antenatal care services in a post-conflict district of  Northern Uganda actively link women to skilled birth attendant services | Fear of being neglected or maltreated by health workers; long distance and other difficulties in access; poverty, and material requirements for delivery; lack of support from husband/partner; health systems deficiencies such as inadequate staffing/training, work environment, and referral systems (poor ambulance services); and socio-cultural and gender issues such as preferred birthing position and preference for traditional birth attendants | 75 |
| 90 | Atuoye et al. (2015) | Ghana | Qualitative study: focus group discussions | Eight (8) focus group discussions involving males (n = 40) and females (n = 45) | Investigate transportation barriers in health access in a rural context based on perceived cause, coping mechanisms and strategies for a sustainable transportation system. | poor road network, poverty, lack of emergency services and transport planning | 75 |
| 91 | Atuyambe et al. (2009) | Uganda | Qualitative study: focus group discussions and key informant interviews | focus group discussions among adolescent  girls (10 to19 years) and key informant interviews with health workers. | To explore adolescent health seeking behavior during pregnancy and early motherhood | Stigma (fear of going to health unit, ashamed on meeting peers), transportation difficulties, Cultural practices & beliefs about births, Lack of decision making power, | 75 |
| 92 | Bayley et al. (2013) | Malawi | Quantitative: Survey | Respondents included 42 nurse midwives, 1 clinical  officer, 4 medical assistants and 5 other staff. | assessed healthcare  providers’ knowledge of management of routine labour, emergency  obstetric care and emergency newborn care; correlated knowledge  with reported confidence and previous study or training; and measured  perception of the care they provided. | Deficits in knowledge of correct monitoring during routine  Labour, management of eclampsia  and pre-eclampsia. | 75 |
| 93 | Bayou and Gacho (2013) | Ethiopia. | Quantitative:  Cross sectional survey | 229 mothers who resided in the villages and gave birth between January 01 to December 31, 2008 were the study subjects. | to assess utilization of clean and safe delivery service and associated factors | Low level of education, low health literacy, not confident in the ability of health extension workers, distance,  health extension workers not available, fear of being badly treated, lack of money, increasing age and parity | 100 |
| 94 | Bayu et al. (2015) | Ethiopia | Quantitative:  a community-based follow-up study | 522 study participants made up of second- and third-trimester pregnant women who had planned for institutional delivery in South Tigray Zone | identify factors affecting unplanned home delivery in urban settings, where there is relatively  good access in principle to modern healthcare institutions | Single motherhood, illiteracy absence of antenatal clinic visit for indexed pregnancy, absence of obstetric complications during the index pregnancy, low autonomy, and absence of birth preparedness and complication readiness were significant predictors of unplanned home birthing. | 100 |
| 95 | Bazzano et al. (2008) | Ghana | Qualitative component | participant observation, interviews, case histories, and focus groups | To examine the social costs to women of skilled attendance at birth in rural Ghana. | Costs of birthing supplies, lack of  confidence in health staff, transportation costs, cultural expectations and beliefs about having a home birth, | 75 |
| 96 | Bedford et al. (2013) | Ethiopia | Qualitative: semi-structured interviews | mothers who had recently delivered (n = 30) or were pregnant (n = 16) | To identify reasons why women who access  health facilities and utilise maternal new-born and child health services at other times, do not necessarily deliver at  health facilities. | Cultural perceptions encouraging home birthing, inability to employ preferred birthing positions, Distance from home to health facility and lack of transport, the possibility of onwards referral and lack of immediate treatment, lack of decision-making power/autonomy, lack of confidence in health professionals | 75 |
| 97 | Cham et al. (2005) | Gambia | Qualitative: maternal death review | Review of 42 maternal deaths of women who actually tried to reach or reached health care services. | To describe the socio-cultural and health service factors associated with maternal deaths in rural Gambia. | underestimation of the severity of the complications, bad experience with the health care system, delay in reaching an appropriate medical facility, lack of transportation, prolonged transportation, seeking care at more than one medical facility and delay in receiving prompt and appropriate care  after reaching the hospital. | 75 |
| 98 | Chapman (2003) | Mozambique | Qualitative: in-depth interviews and longitudinal pregnancy case studies | key informant interviews  with 83 women of reproductive age during pregnancy and after birth, life histories of a subset of 15 women from the  pregnancy case study group, and focus group sessions. | to examine pregnant women’s underutilization of clinic-based prenatal services | Cultural and religious beliefs | 75 |
| 99 | Chi et al. (2015) | Burundi  and Northern Uganda | qualitative comparative case study: semi-structured in-depth  interviews and 4 focus group discussions | Participants were 32  local health providers and 37 staff of non-governmental organizations working in the area of maternal health | to explore the barriers to effective delivery of emergency obstetric and neonatal care services in post-conflict Burundi  and Northern Uganda | Shortage of qualified staff; lack of essential installations, supplies and medications; increasing workload, burn-out and turnover; and poor data collection and monitoring systems. Barriers unique to Northern Uganda were demoralised personnel and lack of recognition; poor referral system; inefficient drug supply system; staff absenteeism in rural areas; and poor coordination among key personnel. In Burundi, weak curriculum; poor harmonisation and coordination of training; and inefficient allocation of resources were the unique challenges | 75 |
| 100 | Cofie et al. (2015) | Ghana | Qualitative: birth narratives | birth narratives of mothers (n = 20) who experienced pregnancy/labor complications, and fathers (n = 18) whose partners experienced such complications in their last pregnancy. | Explored the influence of birth location preference on women’s pregnancy, labor and birth outcomes. | Perceptions of homebirth as a norm  previous experience of homebirths, high costs of traveling to health facilities, and distance to such facilities. | 75 |
| 101 | Conrad et al. (2012) | Uganda | mixed methods: semi-structured interviews, structured observations of provider–patient interactions, and infrastructure assessment  of selected health facilities. | 26 structured client–provider observations; semistructured interviews with 30 pregnant  women and semistructured interviews with all midwives working at the four selected facilities. | appraise the quality of antenatal care services in a rural district of Uganda | ineffective organization of educational sessions; selective omission of certain services; lack of explanation of important clinical and laboratory procedures; failure to link the performed procedures with preventive information; and occasional lack of respect for clients | 75 |
| 102 | Crissman et al. (2013) | Ghana | Qualitative: semi-structured interviews | semi-structured interviews with 85 pregnant women attending an antenatal clinic in Akwatia, | to better understand the barriers to skilled birth attendance and healthcare facility delivery through the perspective of pregnant women. | maltreatment by midwives; cost associated with facility-based delivery despite waived facility fees; the need for a support person for facility-based delivery; difficulties in transportation; and precipitous labour | 100 |
| 103 | Dahlberg et al. (2015) | Kenya | Qualitative: in-depth interviews and focus group discussions | 25 in-depth interviews  with mothers of children under 2 years (13) and healthcare staff (12) and held 10 focus group discussions with traditional birth attendants (6) and female relatives (4)  rural community | To understand how place of childbirth is determined | Over- worked healthcare staff, under-staffed facilities, insensitivity at health facilities (harsh, arrogant, abusive, negligence), influence of female relatives on place of delivery, distance, costs. | 100 |
| 104 | De Allegri et al. (2015) | Burkina Faso | mixed methods: in-depth interviews and household survey | series of open-ended interviews with 55 purposely selected households and 13 village leaders; household survey on 1130 households. | explore reasons for home delivery in rural Burkina Faso, where a successful  user fee reduction policy is in place since 2007. | Lower socio-economic status, distance to the health facility, poor road networks, travel costs, and the cost-sharing fees at health facilities. | 100 |
| 105 | Echoka et al. (2014) | Kenya | Qualitative: In-depth interviews | 30 women who experienced obstetric “near miss” at the only public hospital with capacity to provide comprehensive EmOC services in the district | to  explore barriers to emergency obstetric care (EmOC) services by women who experienced life threatening obstetric complications in Malindi District, | lack of birth preparedness, including failure to identify a health facility for delivery services, and to seek care promptly despite recognition of danger signs. Long distance and inconvenient transport to hospital, lack of money, long waiting times at health facilities and unavailability of doctors | 75 |
| 106 | Essendi et al. (2015) | Kenya | Qualitative: Focus group discussions and key informant interviews | Focus group discussions with mothers and partners and key informant interviews with health care providers and community leaders. In total, 12 focus group discussions (6 in each site) and 4 key informant interviews (2 in each site) were conducted | to understand community and provider perceptions of the obstacles faced in  providing and accessing maternal and newborn care at health facilities in their localities. | Lack of capacity by health facilities to provide around-the-clock services due to inadequate staffing and lack of resources to operate at night; lack of electricity and water; poor roads, | 75 |
| 107 | Essendi et al. (2010) | Kenya | Qualitative: Focus group discussions | Women aged between 12 and 54 years who had a pregnancy outcome in 2004–2005, had life-threatening obstetric complications and failed to seek health care were purposively sampled for focus group discussions. Their partners, opinion leaders, traditional birth  attendants, and older women were also included. 16 focus group discussions were held with each of the groups | to investigate views surrounding  barriers to the uptake of formal obstetric services. | Ineffective health decision making at the family level, inadequate transport facilities and insecurity at night, high cost of health services, and inhospitable formal service providers and poorly equipped health facilities in the slums | 75 |
| 108 | Faye et al. (2011) | Senegal | Quantitative: cross-sectional survey | Three hundred and seventy-three women who gave birth in the last 12months. | To study the link between patients’ satisfaction about received services in  health facilities and the choice of future delivery place of women who had delivered at least once in a facility | Women in a polygamous marriage and those with a parity greater than three were more likely to have a homebirth. Other factors were a lack of means of transport, distance of more than 5km from the health facility, unsatisfactory quality of care, delivery assisted by a man. | 75 |
| 109 | Feinstein et al. (2013) | Congo | Quantitative: cross-sectional survey | 1221 women ≥18 years old who had been pregnant within the prior three years. | To understand where and how women access reproductive healthcare services in Kinshasa. | Low participant and partner education and lack of certain assets, dissatisfaction with provider interactions | 75 |
| 110 | Feyissa and Genemo (2014) | Ethiopia | Quantitative: Retrospective unmatched case control study design | 320 respondents (80 cases and 240 controls). Cases were women who gave birth to their last child in health  institutions in the last five years. Controls were women who give birth to their last child at home in the last five years in East Wollega zone. | to assess determinants of institutional delivery in Western Ethiopia. | Low level of education, long distance to facility, higher parity, rural residence, lack of appropriate means of transport | 100 |
| 111 | Ganle et al. (2014) | Ghana | Qualitative: focus group discussions and in-depth interview | 185 expectant and lactating mothers and 20 healthcare  providers in six communities | To explore health system factors that inhibit women’s access to and use of skilled maternal and new born healthcare services in Ghana despite these services being provided free. | Experiences of intimidation in healthcare facilities, unfriendly healthcare providers, cultural insensitivity, long waiting time before care is received, limited birthing choices, poor care quality, lack of privacy at healthcare facilities, and difficulties in arranging suitable transportation | 100 |
| 112 | Ganle (2015) | Ghana | Qualitative: focus group discussions | 6 Focus group discussions with 94 Muslim women in three communities in northern Ghana | To explore the maternity healthcare needs and care experiences of Muslim women and the barriers to accessing and using maternal health services | a religious obligation to maintain bodily sanctity through modest dressing and the avoidance of unlawful bodily exposure or contact with certain people including male or alien caregivers, a lack of privacy, healthcare providers’ insensitivity and lack of knowledge about Muslim women’s religious and cultural practices, and health information that lacked the cultural and religious specificity to meet Muslim women’s maternity care needs. | 100 |
| 113 | Gebrehiwot et al. (2014) | Ethiopia | Qualitative: in-depth interviews | Twelve in-depth interviews were carried out with eight health extension workers and four midwives. | To explore health-service providers’ perceptions of facilitators and barriers to the utilization of institutional delivery in  Tigray, a northern region of Ethiopia | ‘Delivery as a natural event’, ‘cultural tradition and rituals’, ‘inaccessible transport’, ‘unmet community expectation’ and ‘shortage of skilled human resources’ | 100 |
| 114 | Gebrehiwot et al. (2012) | Ethiopia | Qualitative: focus group discussions | six focus group discussions with 51 women to explore perceptions and experiences regarding delivery care. | To explore women’s experiences and perceptions regarding delivery care in Tigray, a northern region of  Ethiopia, | Transportation difficulties, uncertain quality of care, faith/religious beliefs, influence of older women in the household (preference for home births), distrust of health facilities. | 100 |
| 115 | Grossmann-Kendall et al. (2001) | Benin | Qualitative: in-depth interviews | 19 women aged 20-40 who had recently given birth in a referral hospital | Explore women’s experiences of antenatal and emergency obstetric care | Poor quality of care including not being able to ask questions or get any explanations, being mistreated and humiliated by health personnel | 100 |
| 116 | Habte and Demissie (2015) | Ethiopia | Quantitative: survey | 816 women who gave birth within the past 2 years and  lived in Cheha district for minimum of one year prior to the survey | To measure the prevalence and to identify factors associated with institutional delivery service utilization among childbearing mothers in Cheha District, SNNPR, Ethiopia. | Rural residence, affordability, husband’s negative attitude about facility, non-receipt of counselling on facility delivery during antenatal care for previous pregnancy, | 100 |
| 117 | Kawakatsu et al. (2014) | Kenya | Quantitative: survey | 2,560 women who had children aged 12–24 months. | To identify the factors which influence the place of delivery in rural western Kenya | Low education level, poor health literacy. Low number of antenatal care attendance, long distance to heath facilities | 100 |
| 118 | Kawuwa et al. (2007) | Nigeria | Qualitative: in-depth interviews | Thirty representatives of interest groups including traditional and religious leaders, local government administrators, teachers, civil servants, members of the national union of road transport workers, women community leaders and women non-governmental organizations and staff of the local government primary health care department. | To identify barriers to prompt and effective treatment of obstetric complications leading to maternal mortality | Lack of money, transportation difficulties and community's impression of the health facility's capability to handle their problems. | 75 |
| 119 | Kaye et al. (2014) | Uganda | Qualitative: in-depth interviews | 16 participants - women who developed uterine rupture following obstructed labour. | To explore lived experiences of women who developed uterine rupture following obstructed labour. | Failure to recognise danger signs of obstructed labour, late decision making for accessing care, geographical barriers to health facilities, late or failure to diagnose obstructed labour at health facilities, and failure to promptly perform caesarean section | 75 |
| 120 | Kaye et al. (2000) | Uganda | Qualitative: Participatory observation, midwife and client interviews, records review,  facility assessment and focus group discussions with clients and patients. | Patients admitted in the health units with pregnancy complications; attendants of  patients; midwives delivering health care at the health units; pregnant women  exiting from (after attending) antenatal clinics; and health unit records. | To determine the quality of care provided by midwives; and specifically, to identify training needs, gaps in knowledge and other barriers to accessibility of emergency obstetric care services in Soroti district | Inadequate pre-service and in-service training, lack of technical support supervision and absence of standard treatment guidelines, inability to identify and manage women with or at risk of pregnancy complications. | 75 |
| 121 | Keri et al. (2010) | Uganda | Qualitative: focus group discussions | Six focus groups were held in rural areas surrounding Kampala, the capital city of Uganda | To assess current beliefs, knowledge and practices of Ugandan traditional birth attendants and their pregnant patients regarding referral of obstructed labour and fistula cases | Reported abuse by doctors and nurses, and seeing fistula as a disease caused by hospitals. | 100 |
| 122 | King et al. (2015 | Ethiopia | Qualitative: semi-structured interviews and focus group discussions | Semi-structured  Interviews with health workers, health extension workers and  women. | To explore barriers and facilitators that enable women to access killed birth attendance in  Afar Region, Ethiopia. | Women's low status and restricted opportunities for decision-making, lack of confidence in health-care facilities, long distances, cost, domestic workload, and traditional practices which include a preference for birthing at home with a traditional birth attendant. | 100 |
| 123 | Kujawski et al. (2015) | Tanzania | Quantitative: survey | Women on discharge from delivery at two hospitals. | Assessed the association between reported disrespectful treatment during childbirth and delivery satisfaction, perceived quality of care, and intention to deliver at the same facility in the future. | Disrespect/abuse from health care workers during childbirth | 50 |
| 124 | Kumbani et al. (2013) | Malawi | Qualitative: in-depth interviews | 12 in- depth interviews with women who had delivered at home within the period December 2010 to March 2011 | To explore the reasons why women delivered at home without skilled attendance despite receiving antenatal care at a health centre and their perceptions of perinatal care. | Onset of labour at night, rainy season, rapid labour, socio-cultural factors and health workers’ attitudes. | 75 |
| 125 | Kwagala (2013) | Uganda | Qualitative: in-depth interviews with mothers, focus group discussions with mothers and fathers and key informant interviews. | 10 key-informant interviews with health personnel, TBAs and local leaders; 9 in-depth interviews with selected mothers and 4 focus-group discussions with 10–12 participants each, of  women and fathers (29–46 years) | Examined maternal health-seeking behaviour among the Sabiny people of  Eastern Uganda in relation to health policy ideals | Limited decision-making power of women, cultural ideals associated with enduring pain (an endurance test and the marker of a real woman), lack of responsible persons with whom to leave older children, transportation challenges and poor roads, preference for TBA-assisted deliveries and the desire/need to follow local birthing and post-delivery practices, miscalculation of delivery date and a lack of waiting space at the health facilities for women in labour., inability to afford services | 75 |
| 126 | Kyomuhendo (2003) | Uganda | Mixed methods  study: focus group discussions, key informant  interviews, a quantitative survey and maternal  death enquiries | 24 focus group discussions involving 240 participants (with men only and women only in each of five localities); 808 women with  more than one birthing experience were interviewed  in the quantitative survey; maternal death inquiries  with relatives, kin of the  deceased or the birth attendants of women who died of maternal causes in the community in the last 1–12 months preceding study | To enhance understanding of why, when faced with  complications of pregnancy or delivery, women continue to choose high risk options leading to  severe morbidity and even their own deaths. | Adherence to traditional birthing practices and beliefs that pregnancy is a test of endurance, perception that maternal death a sad but normal event, illiteracy, poverty, a lack of skilled staff at primary health care level, complaints of abuse, neglect and poor treatment in hospital and poorly understood reasons for procedures, health workers’ views that women were ignorant, | 75 |
| 127 | Lerberg et al. (2014) | Gambia | Quantitative: survey | 432 women of reproductive age (15-49 years), living in North Bank East Region, who had given birth outside a health facility within the last six months prior to the day of data collection, | to identify the most important barriers for use of skilled attendance during childbirth by women in rural Gambia. | A lack of time, lack of transport, poor services at facilities, childbirth occurring before arrival of transport, poor roads. | 75 |
| 128 | Magoma et al. (2010) | Tanzania | Qualitative: key informant interviews and focus group discussions | Twelve key informant interviews and fifteen focus group discussions (key stakeholders in maternal health, users of antenatal, delivery, and post-delivery care, 36 total traditional birth attendants and 16 elders | Examined beliefs and behavioUrs related  to antenatal, labor, delivery and postnatal care among the Maasai and Watemi ethnic groups | Distance from health units, lack of reliable and affordable transport, lack of advanced planning for accessing delivery care units, widely held beliefs that pregnancies labelled as ‘normal’ during antenatal care visits will result in successful deliveries at home, failure of providers to convey information about the importance of skilled delivery care for all women, and women’s low social status and inability to independently make labour and delivery decisions. | 100 |
| 129 | Mahiti et al. (2015) | Tanzania | Qualitative: focus group discussions | 15 focus group discussions with women attending a health facility after child birth | To explore women’s views about the maternal health services (pregnancy, delivery, and postpartum period) that they received at health facilities in order to identify gaps in service provision that may lead to low-quality maternal care and increased risks associated with maternal  morbidity and mortality | availability and use of traditional birth attendants, long distances to facilities, shortage of maternal health workers, long waiting times and informal payments at health facilities, drug shortages, dirty health facility environment. | 100 |
| 130 | Mills and Bertrand (2005) | Ghana | Qualitative: focus group discussions | Eighteen purposively sampled homogenous groups in Kassena-Nankana  District of northern Ghana participated in focus-group discussions | Explored the role of access versus traditional beliefs in the decision to seek obstetric care from health professionals | Late decision-making about place of delivery (generally after the onset of labour), cost of care, distance, transport, availability of health facilities, and nurses’ attitudes | 100 |
| 131 | Mkoka et al. (2014) | Tanzania | Qualitative: in-depth interviews | 17 health facility managers (14 from dispensaries and  three from health centers); two members of the Council Health Management Team and one member of the Council Health Service Board | To describe the experience of rural health facility managers in ensuring the  timely availability of drugs and medical supplies for emergency obstetric care | Unreliability of obtaining drugs and medical supplies due to insufficient budget for drugs from central government, lack of accountability within the supply system and a bureaucratic process of accessing the locally mobilized drug fund | 100 |
| 132 | Mselle et al. (2013) | Tanzania | Qualitative: Semi-structured interviews and  focus group discussions | Semi-structured interviews involving 16 women affected by obstetric fistula and five nurse-midwives at  maternity wards, and focus group discussions with husbands and community members | To describe the weaknesses in the  provision of acceptable and adequate quality care through the accounts of women who have suffered obstetric fistula, nurse-midwives at both basic and comprehensive EmOC health facilities and local community members | Health providers experienced dissatisfaction with the working environment, lacked supportive supervision, seemed to lack motivation, as well as inadequate supplies  Women in labour lacked support, experienced neglect, as well as physical and verbal abuse at health facilities, lack of trust in the health facility. | 100 |
| 133 | Mwangome et al. (2012) | Kenya | Qualitative: individual interviews and focus group discussions | Twelve discussion groups were with hospital staff and general community members (36 males and 54 females), individual interviews with 26 mothers who chose not to deliver their babies in hospital. | Identified attitudes to and  beliefs about the uptake of hospital services for birthing. | A lack of financial resources to cover cost of services, a lack of means of transport, geographic distance to facility, fear of being verbally abused by hospital staff, fear of caesarean delivery, not being allowed to assume preferred positions during birthing, negligence by nurses, fear of being tested for HIV lack of knowledge about pregnancy and maternal health), negative influence of relatives. | 75 |
| 134 | Ng’anjo Phiri et al. (2014) | Kenya, Tanzania and Zambia | Quantitative: survey | 1800 women who had childbirth in the previous five years | To investigate the underlying and proximate determinants of health facility childbirth in rural and urban areas of three districts in Kenya, Tanzania and Zambia. | Perceived quality and trust of local health services, perceived distance to facility | 75 |
| 135 | O’Donnell et al. (2014) | Malawi | Qualitative: in-depth interviews and focus group discussions | 27 in-depth interviews and 2 focus group discussions with 33 postnatal mothers and 10 healthcare providers from all 4 major hospitals in one district | To examine perceptions of the quality of care provided during childbirth in Malawi | Lack of autonomy and decision making power is a barrier to  quality of care and it exists both at the level of the patient (mother) and at the level of her caregiver with  healthcare providers unable to influence decisions made by more senior staff or management. Lack of autonomy and demotivation, frustration, lack of empowerment to make change, resulting in a poor quality of care provided. | 75 |
| 136 | Oiyemhonlan et al. (2013) | Ghana | Mixed methods: survey, in-depth interviews and focus group discussions | 2 emergency obstetric cases, 29 antenatal focus group discussants and 5 midwives at the maternity unit. | To identify obstetric emergencies and barriers to emergency care seeking; examine the perspective of midwives regarding their role in maternity care and management of obstetric emergencies, and explore women’s knowledge and response to obstetric  emergencies. | At the individual level, obstetric complications were poorly understood by antenatal women, there were also lack of readily accessible transportation, poverty, high illiteracy rates language and cultural barriers. Service challenges included insufficient staffing, inadequate equipment and physical space in the maternity ward. | 50 |
| 137 | Okafor et al. (2014) | Nigeria | Qualitative: focus group discussions | focus group discussions.  with women of reproductive age group within a rural Local Government Area  in Lagos state. | To determine the use of orthodox versus unorthodox maternity healthcare and determinants among rural women in southwest  Nigeria. | Traditional beliefs and practices, a lack of financial resources, | 75 |
| 138 | Ono et al. (2013) | Kenya | Quantitative: survey  Facility-based | 303 mothers who brought their babies to the health center for immunization within their first year of life. | To explore determinants of association between social support and place of delivery. | Being married, unmarried women without support in housework from family (mother or sisters), married women with support from husbands and neighbours, not receiving advice on health facility delivery from mother-in-law or health staff | 50 |
| 139 | Osubor et al. (2006) | Nigeria | Mixed methods  study: survey and focus group discussions | 225 randomly selected mothers (age 15–49 years), six focus group discussions — four for community  women and two for health workers | To assess maternal health services and health-seeking behaviour in a rural community | Irregularity of staff at work, poor quality of services, ignorance about warning signs in pregnancy, and preference for traditional birth attendants due to greater accessibility, better interpersonal relationship, lower cost, greater convenience, and freedom to use traditional birthing  positions, | 75 |
| 140 | Pettersson et al. (2004) | Angola | Qualitative: focus group discussions | Ten focus group discussions with pregnant and non-pregnant women residing in suburban areas of Luanda | To explore how various factors influenced women’s decisions regarding place of confinement in Luanda, Angola. | Demand for informal user fees, perceived low quality of care, dissatisfaction with services of midwives and obstetricians, women’s inability to choose their positions during delivery, impact of socio-psychological effects of migration from war-affected areas, shame of poverty, family’s influence to adhere to traditional practices, reluctance by midwives to care for complicated cases, women’s own decision to avoid institutional care. | 100 |
| 141 | Ridge et al. (2010) | Zambia | Qualitative: observation and reords review | A ‘fishbone’ (Ishikawa) diagram listing probable facilitators to the availability and use of MgSO4, a walk through observational exercise and a review of available undergraduate medical and midwifery education and training materials | To identify barriers to the availability and use of MgSO4 in the Zambian Public Health  System. | Lack of procurement by the Ministry of Health, a lack of demand by health professionals at the health centre level and a lack of in-service training in the use of MgSO4. Where there was demand by obstetricians, magnesium sulphate injection was being procured from the private sector by the hospital pharmacy despite not being registered and licensed for use for the treatment of severe pre-eclampsia and eclampsia by the  national Pharmaceutical Regulatory Authority. | 100 |
| 142 | Roka et al. (2013) | Kenya | Quantitative: case control study | Seventy cases and 140 controls were included in the study (Cases were patients who had fistula following delivery within the previous five years. Controls were systematically selected from women who attended obstetrics and gynaecology clinics at these hospitals, and did not have present or past history of fistula.) | To identify risk factors associated with developing obstetrics fistula in order to guide implementation of appropriate interventions | Delays in care seeking including delay in making  decision to seek delivery servers after six hours of labour onset, taking more than two hours to reach a health  facility, and having no formal or primary education. | 100 |
| 143 | Roro et al. (2014) | Ethiopia | Qualitative: focus group discussions | Eight focus group discussions - four with women and four with men groups were conducted involving 81 residents of the Butajira district | To make an in-depth assessment of reasons why mothers do not use health facilities for child delivery. | A lack of decision-making power on place of delivery, reliance on traditional birth attendants, misconception about services provided at health facility, inability of family members to be present at time of labour and delivery, lack of privacy, traditional and/or spiritual factors, economic factors and accessibility to health care facilities, poor reception at health facility, refusal of admission, information gap, poor competence and shortage of staff and materials at health facilities | 100 |
| 144 | Schack et al. (2014) | Ghana | Qualitative: in-depth interviews | Twelve in-depth interviews were conducted with labor ward midwives who all had previous training  in active management of the third stage of labour  Facility-based | To gain an in-depth understanding of midwives’ experiences about active management of the third stage of labour | Knowledge gaps in Active management of the third stage of labour protocol, insufficient staff overage, | 100 |
| 145 | Seljeskog et al. (2006) | Malawi | Qualitative: in-depth interviews and non-participatory observation | Six women who had given birth (three at home and three in a health facility) | To determine factors influencing women’s choice of place of delivery | Sub-optimal quality of care such as poor communication, poor attitudes; long distance to facility, poor access to transportation, costs, influence from decision-makers, perception of danger signs, traditional views on pregnancy and birthing. | 100 |
| 146 | Sialubanje et al. (2015) | Zambia | Qualitative: in-depth interviews and focus group discussions | Ten focus group discussions (n = 100) with women of reproductive age (15–45 years) and 30 in-depth  interviews were conducted comprising 5 traditional birth attendants, 4 headmen, 4 husbands, 4 mothers, 4 neighbourhood health committee members, 4 community health workers and 5 nurses. | To identify reasons motivating women to giving birth at  home and seek the help of traditional birth attendants. | Women’s lack of decision- making autonomy regarding child birth, dependence on the husband and other family members for the final decision, and various physical and socioeconomic barriers including long distances, lack of money for transport, the requirement to bring baby clothes and food while staying at the clinic, socio-cultural norms regarding childbirth, and negative attitude towards the quality of services provided at the clinic | 100 |
| 147 | Singh et al. (2015) | Ghana | Mixed methods: survey, in-depth interviews and focus group discussions | Household survey (n = 1267 women), a quantitative  community leader survey (n = 62), qualitative birth narratives with mothers (n = 20) and fathers (n = 18), key  informant interviews with health care workers (n = 5) and focus groups (n = 3) with community leaders and  stakeholders. | To describe women’s experiences with the National Health Insurance Scheme and to study associations between insurance and skilled facility delivery, antenatal care and early care-seeking  for sick children. | Transportation barriers, fear of unknown facilities, expenses not covered or believed to be not covered by insurance, extreme poverty, low level of education, challenges regarding registration for the health insurance, and lack of understanding of who and what services were covered for free. | 100 |
| 148 | Somé et al. (2014) | Burkina Faso | Qualitative: in-depth interviews and focus group discussions | Thirty in-depth interviews, 8 focus group discussions and 6 non-participant observations were carried out. Participants were women from 15-49 years. | To describe barriers which  prevent women from sustainable use of maternal care | Cultural barriers (low status of women, traditional beliefs, women’s low decision-making power, distance to health facility, lack of transport means, inability to afford service and drugs costs, and poor quality of care provided to women | 75 |
| 149 | Sorensen et al. (2011) | Tanzania | Mixed methods: survey and in-depth interviews | Survey and semi-structured in-depth interviews were carried out with 97 providers and users of delivery care for 31 births at home, at village-based health facilities and at Kagera Regional Hospital | To analyse the main dynamics and conflicts in attending and providing good quality delivery care in a local Tanzanian rural setting | Long distance to facilities, availability of means of transport costs, poor quality of care | 50 |
| 150 | Speizer et al. (2014) | Ghana | Quantitative: survey | 1,606 includes  all women who had a birth three years prior to the survey date and who had no missing data. | To examine the psychological and  social barriers to institutional delivery, namely women’s decision-making autonomy and their perceptions about social  support for institutional delivery in their communities | Women’s low decision-making autonomy, | 100 |
| 151 | Storeng et al. (2007) | Burkina Faso. | Mixed methods  study: in-depth interviews and cohort study | ethnographic study of 82 women nested in a prospective cohort study of  1013 women | To compare the experiences of women who survived life-threatening obstetric complications (‘near-miss’ events) with women who delivered without complications in hospitals. | High cost of emergency obstetric care, low socioeconomic status households. | 75 |
| 152 | Idris et al. (2006) | Nigeria | Quantitative:  survey | 496 women who had delivered at lest once | To assess the role of some health, socio-economic and demographic factors in determining the place of delivery among women in a semi-urban setting | Low mother’s educational status, father’s unemployment status, age of first pregnancy before  18 years | 100 |
| 153 | Oguntunde et al. (2010) | Nigeria | Quantitative:  survey | 332 women who had delivered within two years of the survey | assessed antenatal care (ANC) coverage, place of delivery and use of skilled birth assistants in three communities in Kaduna State | Low educational status, poor quality and coverage of antenatal and birthing services | 100 |
| 154 | Ejembi et al. (2004 | Nigeria | Quantitative:  survey | 655 married women of reproductive years (10 to 49 years) | To document level and pattern of utilisation of selected maternal health services among Hausa women | Perception that antenatal care was not necessary, husband’s refusal, being unaware of services, culturally unacceptable hospital practices, geographical inaccessibility, high costs of services, negative staff attitudes. | 100 |
| 155 | Moyer et al. (2013a) | Ghana | Qualitative: in-depth interviews and focus group discussions | 72 in-depth interviews with mothers, Traditional Birth Attendants, herbalists, other local healers, nurses, midwives, medical assistants, medical doctors. and 18 focus group discussions with grandmothers, household heads, and compound heads | To explore the impact of social factors on place of delivery in northern Ghana | traditional religions, illiteracy, requiring permission before traveling to a health facility, Lower socioeconomic status, difficulties obtaining transportation, the cost of transportation, and the cost of care-seeking | 100 |
| 156 | Moyer et al. (2013b) | Ghana | Qualitative: in-depth interviews and focus group discussions | Focus groups and 43 individual interviews were conducted with community members, and 13 individual interviews were conducted with healthcare providers | To explore community and healthcare provider attitudes towards maltreatment during  delivery in rural northern Ghana and compare findings against The White Ribbon Alliance's seven  fundamental rights of childbearing women. | Physical abuse, verbal abuse, neglect, discrimination and denial of traditional practices. | 100 |
| 157 | Lori and Boyle, (2011) | Liberia | Qualitative: in-depth interviews and participant observation | Semistructured, in-depth interviews with postpartum women who experienced a maternal complication, community and family members of women who died from a pregnancy or childbirth complication.  Participant observation was used to understand and interpret cultural behavior | To provide an understanding of childbirth and maternal illness and death through the lens of women, communities, and families | Lack of decision-making autonomy, distrust of the health care system, fear of routines or procedures of the hospital | 100 |
| 158 | Izugbgara et al. (2009) | Kenya | Qualitative: focus group discussions | 12 focus group discussions involving 74 purposefully selected women from two slums in Nairobi | To investigate the attractions of and deterrents to hospital-based deliveries | Poverty, cost of services, transportation cost, harsh and uncaring attitudes of hospital-based providers, fear of HIV testing, providers lack the requisite training and skills | 100 |
| 159 | Mrisho et al. (2007) | Tanzania | Mixed methods: In-depth interviews, focus group discussions, participant observation and survey | Thirty-two in-depth interviews, 2 focus group discussions, participant observation and survey of 21,600 randomly chosen households (all women aged 15–49 years were asked about all children born in the 3 years prior to the survey) | Combines an understanding of gender issues relating to health and help-seeking behaviour with epidemiological knowledge concerning place of delivery. | A lack of money to pay for delivery kits, transport fare and food, lack of means of transport, sudden onset of labour or short labour, poor staff attitude including abusive language, denying women service, lacking compassion and refusing to assist properly, lack of privacy, traditional beliefs and culture, low level of education, ethnicity, older maternal age | 75 |
| 160 | Stekelenburg et al. (2004) | Zambia | Mixed methods: survey, focus group discussions and records review | 332 women interviewed using semi-structured questionnaires. Focus group discussions were held and hospital data and registers were checked. | To determine the level of use of maternal health services and to identify and assess factors that influence women's choices where to deliver in Kalabo District, Zambia | Long distances, lack of transport, user fees, lack of adequate health education given during antenatal clinic attendances, poorly staffed and ill-equipped institutions with poorly skilled personnel. | 75 |
