# Supplementary material for: Access barriers to obstetric care at health facilities in sub-Saharan Africa—a systematic review
Source: Syst Rev. 2017 Jun 6;6:110. doi: 10.1186/s13643-017-0503-x (PMC5461715; doi:10.1186/s13643-017-0503-x)
Supplement: Supplementary file 5 — PRISMS 2009 Flow Diagram. Flow chart of data extraction process. (DOC 64 kb) [file 13643_2017_503_MOESM5_ESM.doc]

**Additional file 5: Figure S1: Study selection and elimination**

Identification

Eligibility

Screening

Records identified through database searching
(n = 2,974)

Additional records identified through other sources
(n = 63)

Records after duplicates removed
(n = 2,766)

Records screened
(n = 2,766)

Records excluded
(n = 2,381)

Full-text articles assessed for eligibility
(n = 385)

Studies included in review
(n = 160)

Full-text articles excluded, with reasons (n = 225)

- secondary data (n = 61)
- primary outcomes other than obstetric care barriers (n = 98)
- full text not available (n = 9)
- combination of data from outside sub-Saharan Africa (n = 57)

Included
